# Retained particle surface area dose drives inflammation in rat lungs following acute, subacute, and subchronic inhalation of nanomaterials

**DOI:** 10.1186/s12989-021-00419-w

**Published:** 2021-08-05

**Authors:** Frédéric Cosnier, Carole Seidel, Sarah Valentino, Otmar Schmid, Sébastien Bau, Ulla Vogel, Jérôme Devoy, Laurent Gaté

**Affiliations:** 1 Institut National de Recherche et de Sécurité, 1 Rue du Morvan, CS 60027, 54519 Vandœuvre-les-Nancy Cedex, France; 2 Institute of Lung Biology and Disease, Helmholtz Zentrum München, 85764 Neuherberg, Germany; 3 Comprehensive Pneumology Center, Munich (CPC-M) - Member of the German Center for Lung Research (DZL), 81377 Munich, Germany; 4 National Research Centre for the Working Environment, Lersø Parkallé 105, DK-2100 Copenhagen, Denmark; 5 Department of Health Technology by DTU Food, Technical University of Denmark, DK-2800 Kgs. Lyngby, Denmark

**Keywords:** Inhalation, Aerosol, Rat, Titanium dioxide, Carbon black, Multiwall carbon nanotube, SBET, Neutrophil influx, Retained surface area, MPPD

## Abstract

**Background:**

An important aspect of nanomaterial (NM) risk assessment is establishing relationships between physicochemical properties and key events governing the toxicological pathway leading to adverse outcomes. The difficulty of NM grouping can be simplified if the most toxicologically relevant dose metric is used to assess the toxicological dose-response.

Here, we thoroughly investigated the relationship between acute and chronic inflammation (based on polymorphonuclear neutrophil influx (% PMN) in lung bronchoalveolar lavage) and the retained surface area in the lung. Inhalation studies were performed in rats with three classes of NMs: titanium dioxides (TiO_2_) and carbon blacks (CB) as poorly soluble particles of low toxicity (PSLT), and multiwall carbon nanotubes (MWCNTs). We compared our results to published data from nearly 30 rigorously selected articles.

**Results:**

This analysis combined data specially generated for this work on three benchmark materials - TiO_2_ P25, the CB Printex-90 and the MWCNT MWNT-7 - following subacute (4-week) inhalation with published data relating to acute (1-week) to subchronic (13-week) inhalation exposure to the classes of NMs considered. Short and long post-exposure recovery times (immediately after exposure up to more than 6 months) allowed us to examine both acute and chronic inflammation.

A dose-response relationship across short-term and long-term studies was revealed linking pulmonary retained surface area dose (measured or estimated) and % PMN. This relationship takes the form of sigmoid curves, and is independent of the post-exposure time. Curve fitting equations depended on the class of NM considered, and sometimes on the duration of exposure. Based on retained surface area, long and thick MWCNTs (few hundred nm long with an aspect ratio greater than 25) had a higher inflammatory potency with 5 cm^2^/g lung sufficient to trigger an inflammatory response (at 6% PMN), whereas retained surfaces greater than 150 cm^2^/g lung were required for PSLT.

**Conclusions:**

Retained surface area is a useful metric for hazard grouping purposes. This metric would apply to both micrometric and nanometric materials, and could obviate the need for direct measurement in the lung. Indeed, it could alternatively be estimated from dosimetry models using the aerosol parameters (rigorously determined following a well-defined aerosol characterization strategy).

**Supplementary Information:**

The online version contains supplementary material available at 10.1186/s12989-021-00419-w.

## Background

Exposure to particles is an issue in everyday life and occupational health. Indeed, inhalation of particles and in particular of nanomaterials (NMs) may induce many pulmonary adverse outcomes (AO) [1].

The recently completed EU-funded Smartnanotox project (www.smartnanotox.eu) leveraged data from in vivo, in vitro and in silico studies, and proposed pulmonary AO pathways (AOPs) for inhaled NMs, presenting their associated molecular initiating events or key events (KEs). This structured AOP-based approach for hazard grouping is now considered a relevant tool to assess the risks associated with inhaled materials, particularly for NMs [1, 2].

Both inflammation and oxidative stress are central mechanisms driving NM-induced adverse effects [3]. The extent of pulmonary inflammation depends on the amount of inhaled NM deposited in the lung, in particular in deep lung (i.e., the alveolar region), since mucociliary particle clearance rapidly and effectively clears the upper airways of deposited particles [4]. Particles deposited in the lung remain either for only a short duration (acute), as they are gradually eliminated over time due to various clearance mechanisms, or persist long-term, in which case they can induce chronic inflammation leading to pathologies such as chronic obstructive pulmonary disease (COPD), emphysema, lung fibrosis, or cancer [5, 6].

One hallmark or KE of the inflammatory response in the lungs is the increased recruitment of circulating inflammatory cells [7, 8]. The influx of inflammatory leukocytes, especially polymorphonuclear neutrophils (PMN) which play a major role in the pathogenesis of many respiratory diseases [9], can be assessed using bronchoalveolar lavage (BAL). This method involves rinsing the epithelial surface of the lung with saline solution, the number of PMNs present in the recovered fluid (BALF) can then be counted [10].

As part of a drive to standardize risk assessment or predict risks association with new NMs, research seeks to establish relationships between the physicochemical properties of NMs and the KEs triggering the toxicity pathway leading to AO. Based on these relationships, descriptors can be identified to group NMs according to their toxicological mode-of-action. The difficult task of NM grouping can be substantially simplified if the most toxicologically relevant dose metric is used to measure the toxicological dose-response [11, 12].

In the past decade, growing evidence suggests that deposited or retained particle (NM) surface area normalized to lung mass can be leveraged to unify in vivo inflammation data from mice and rats, based on PMN numbers normalized to total cell numbers in the BALF, and to identify distinct classes of NM. Representatives of some of the different NM classes include titanium dioxide (TiO_2_) [12, 13], multiwall carbon nanotubes (MWCNTs) [14–16], nano-ceria [17], granular biodurable particles and transition metal oxides [12, 18–20], diesel exhaust particles [21], nanoclays [22], and halloysite nanotubes [23].

In the present study, we thoroughly investigated the relationship between inflammation and the lung retained surface area dose for three classes of NMs – TiO_2_, carbon blacks (CBs) and MWCNTs – following inhalation in rats. TiO_2_ and CBs are generally considered granular *PSLT (Poorly Soluble particles of Low Toxicity)* [12, 24], whereas MWCNTs are high aspect ratio nanomaterials (HARN).

Data specifically generated for 4-week exposure to three benchmark materials (TiO_2_ P25, CB Printex-90, and MWCNT MWNT-7 (Mitsui-7)) were combined with data from nearly 30 published studies covering a range of exposure durations (1 week to 13 weeks) and post-exposure recovery times (from immediately after exposure up to more than 6 months) to elucidate the effects of dose and dose rate on both acute and chronic lung inflammation.

## Materials & methods

Analysis combined both newly-acquired data from our laboratory using three benchmark materials administered by subacute (4-week) nose-only (NO) inhalation – the protocol for which is detailed in paragraphs 2.1 to 2.4 – with data from 27 rigorously selected articles reporting acute (1-week) to subchronic (13-week) inhalation of the classes of NMs considered – these studies are detailed in paragraph 2.5.

### Materials & characteristics

The three benchmark materials - TiO_2_ P25, the CB Printex-90 and the MWCNT MWNT-7 (Mitsui-7) - are representatives of the three classes of NMs. These materials were selected as there is abundant literature data related to them. All three are classified in group 2B (“possibly carcinogenic to humans”) by the International Agency for Research on Cancer [25, 26], and induce lung cancer following chronic inhalation in rats [27, 28].

TiO_2_ P25 (Aeroxide® P25) was purchased from Evonik. It is a pure (99.9% TiO_2_) mixed-phase nanocrystalline powder composed of 87% anatase and 13% rutile crystallites, with average primary particle diameters of 21 ± 1.5 nm and 40 ± 1.5 nm, respectively [29]. Printex® 90 (a furnace carbon black from Evonik) and MWNT-7 (Mitsui-7 MWCNT supplied by Mitsui Company) were kindly donated by Mitsui Company via the National Research Centre for the Working Environment (Copenhagen, Denmark). Average particle size of Printex-90 is 14 nm, with purity of around 99% carbon. MWNT-7 is a long (5.0 ± 4.5 μm) and thick (diameter = 88 ± 5 nm) MWCNT composed of 99% carbon.

Gas (N_2_) adsorption measurements performed on the batches of P25, Printex-90, and MWNT-7 used in this study yielded specific surface areas of 55, 316 and 15 m^2^/g, respectively, according to the BET model. Since these materials are not micro or mesoporous, these surface areas correspond to the outer surface of the particles.

### Animal care and exposure

Animal experiments were performed in accordance with European Union Directive 2010/63/EU and with French regulations related to the protection of animals used for scientific purposes, and were conducted in a laboratory animal facility accredited by the French Ministry of Agriculture (Accreditation No D54–547-10). Experimental procedures were approved by the local Ethics Committee and registered by the French Ministry for Research and Higher Education (Authorization n°00692.01 & APAFIS#10052).

Ten-week-old female Sprague-Dawley rats were purchased from Janvier Labs (Le Genest Saint Isle, France). Rats were housed in individually ventilated cages (GR900, Tecniplast) maintained in 12 h/12 h light/dark cycles, and when not in restraining tubes had ad libitum access to food (A04 Safe diet) and water. Two weeks before nose-only exposure to NM aerosols, rats were gradually acclimatized to the restraining tubes.

Animals (from 13 weeks old; 6 rats per group) were then nose-only exposed to either filtered air or NM aerosols (conditioned at 22 ± 2 °C and a relative humidity of 55 ± 10%, in line with the OECD TG 412 guideline [30]) for 6 h/day, 5 days/week for 4 weeks. At least three dose groups were tested for each NM. High dose groups were exposed daily for 6 h to the target concentration: 15, 50, and 1.5 mg/m^3^ for P25, Printex-90, and MWNT-7, respectively. Based on the *Concentration × Time (C× t) protocol* [31], assuming a similar time-dependent lung deposition of the nanostructured aerosols, the medium and low dose groups (expressed as 6 h-equivalent concentrations) were created by modulating the time that animals were exposed to aerosols and assuming a similar time-dependent lung deposition. Exposure to 5 and 15 mg/m^3^ Printex-90 and 0.5 and 0.15 mg/m^3^ MWNT-7 was achieved by exposing rats for 120 and 36 min daily to the target aerosol concentrations (50 and 1.5 mg/m^3^, respectively).

To investigate acute effects of TiO_2_, in addition to the 5 and 1.5 mg/m^3^ groups, three additional groups (10 rats per group) were exposed for 2 weeks to the same concentration levels (or 6 h-equivalent levels): 15, 5, and 1.5 mg/m^3^.

Finally, to observe any effects due to differences in agglomeration states in TiO_2_ aerosols produced from the same starting material, we exposed 13-week-old male Fisher F344 rats (Charles River Laboratories, France) to a 5 mg/m^3^ suspension of TiO_2_ P25 for 6 h/day, 5 days/week for 4 weeks.

Respiratory parameters of control and exposed rats were monitored using plethysmography systems (from Electro-Medical Measurement Systems, Bordon, UK) before, during and after the inhalation exposures. The animals were inside head-out plethysmographs (put directly on the inhalation towers) to measure (once a week) the thoracic flow during exposure and to access the following parameters: tidal volume, inspiration and expiration times, peak inspiratory and expiratory flows, breathing frequency, minute volume and end inspiratory and expiratory pauses. In addition, double-chamber plethysmographs were used the week preceding the exposures or the day preceding the necropsy of the animals to measure nasal and thoracic flows allowing evaluation of the Specific Airway Resistance.

TiO_2_ aerosols were produced from powder using a rotating brush aerosol generator (RBG1000, PALAS, Karlsruhe, Germany) [32] or from a suspension (100 mg/L) in ultrapure water using two nebulizers operated in parallel (AGK2000, PALAS, Karlsruhe, Germany). Printex-90 aerosol was generated using an SAG410/U solid aerosol generator (TOPAS, Dresden, Germany); and MWNT-7 aerosol was produced by an upgraded (high-pressure version) of an acoustic generator (IEStechno, Morgantown, USA) [14, 33] (Supplemental 1 and 2).

Details of the inhalation exposure set-up and the strategy used to characterize and monitor aerosols have been previously described [32]. Briefly, aerosol monitoring relied on the use of (1) a condensation particle counter (CPC) (TSI, model 3007, Shoreview, Minnesota, USA) for the on-line measurement of total submicron particle concentrations, (2) an optical particle counter (OPC) (FIDAS mobile, PALAS, Karlsruhe, Germany) to monitor the airborne particle number size distribution, and (3) systematic closed-face cassette samplers (CFC equipped with PVC or PTFE membranes filters, Millipore, Molsheim France) changed two to four times per day to measure the average mass concentration of the aerosol by gravimetry (XP6U, Mettler-Toledo, Viroflay, France – 0.1 μg resolution). The in-depth characterization of relevant aerosol parameters is described in the same reference [32]. Briefly, it was achieved using time-resolved instruments such as scanning mobility particle sizer (SMPS) (Differential Mobility Analyzer TSI 3082 + Water-based CPC TSI 3787, Shoreview, Minnesota, USA), aerodynamic particle sizer (APS) (TSI 3321, Shoreview, Minnesota, USA), or electrical low-pressure impactor (ELPI, Dekati, Finland). The need for multiple direct-reading instruments is due to the wide range of particle diameters to be covered, typically from 10 nm to 20 μm. The size range covered by each instrument should be addressed during experiment design to ensure appropriate ranges are included. This adaptation is particularly important when further data merging is applied to yield a continuous distribution over the whole range. Time integrated sampling (using SIOUTAS or DLPI+ cascade impactors for example) for a posteriori aerosol characterization is also very important, not only to characterize the aerosols produced in accordance with standard ISO 13014 [34], but also to leverage standard computational lung dosimetry models to estimate the particle dose retained.

### Necropsy, tissue sampling

Lung samples were collected from animals 3, 30 or 180 days (D3, W4 and W26) after the end of the inhalation exposure. Two additional post-exposure times, D0 and W13 (immediately following the last day of exposure, and 90 days later), were also considered with nebulized P25 inhalation exposure to allow comparison with data previously published by our group relating to agglomerated P25 [35, 36]. Animals were anesthetized by intraperitoneal injection of a mixture of xylazine (10 mg/kg body weight) and ketamine (75 mg/kg body weight), then euthanized by exsanguination through the abdominal aorta. After ligation, lung tissue was collected, sectioned, weighed; some lobes were snap frozen in liquid nitrogen and stored at − 80 °C until further analysis.

### Analysis of bronchoalveolar lavage fluid (BALF) and NM lung burden

Following inhalation exposure, deposition is assumed to be homogeneous throughout the lung (no difference between lobes) [37]. BAL was performed on the left lung as described in [14, 35]. Left lungs were flushed 5 times with 4 mL of ice-cold PBS and the pooled BAL fluids were centrifuged 5 min at 4 °C at 400 *g*. Cells from cell pellets were counted using acridine orange - propidium iodide with the Cellometer™ (Nexcelom) and May-Grünwald-Giemsa staining was performed on cytospin slides. Macrophages, PMN and lymphocytes were counted (500 cells/animal) and the %PMN was calculated from the ratio of neutrophils to total cells in BALF. The right median lobe was frozen and used to quantify the NM lung burden.

The TiO_2_ lung burden was determined from elemental Ti analysis by ICP-MS, as previously described [36, 38]. The MWCNT and CB lung burdens (for Printex-90 samples or samples containing short and thin MWCNTs) were quantified by thermogravimetric analysis (TGA) of lyophilized samples after chemical digestion of the tissues with a water-based tissue solubilizer (Solvable, Perkin-Elmer) [39]. The NM surface area retained (in cm^2^/g lung) was calculated for each rat from the retained mass and the wet lung weight (in mg/g lung) combined with the mass-specific BET surface area determined for each material.

### Selection of published studies

In addition to the nose-only inhalation studies described above, more than 50 studies (published before May 2020) relating to pulmonary toxicity of TiO_2_, CB and/or MWCNT after inhalation exposure and using rat as animal model were screened; 27 of them were selected based on the following criteria:

i) Exposure was by whole-body (WB), head-only (HO) or nose-only (NO) inhalation.
ii) Exposure was acute (few days), subacute (4 weeks), or subchronic (13 weeks). Studies describing chronic exposure (2 years) were omitted to avoid the emergence of biological regulation mechanisms specific to long-term exposures.
iii) Results included data on % PMN (polymorphonuclear cells) or PMN and total cell number in the BALF for at least one post-exposure time.
iv) Lung burden (retained mass dose and lung mass) and how it was measured was reported. Alternatively, accurate information was provided (or available) on the aerosol characteristics (Count Median Diameter (CMD), Mass Median Aerodynamic Diameter (MMAD) and corresponding Geometric Standard Deviation (GSD), actual mass concentration, etc.) as well as animal strain, sex and biometry (at least body and lung weight) to allow calculation of the retained mass using the Multiple-Path Particle Model (MPPD) dosimetry model (cf. § 2.6).
v) Accurate physicochemical information was available on the powder used: diameter (and length) of primary particles (tube), chemical purity, crystallographic form if applicable, and most importantly mass-specific BET surface area for conversion of the pulmonary NM mass dose into a surface area dose.

### Estimating pulmonary retention

Pulmonary deposition and retention after inhalation were estimated using the MPPD model (v.3.04), applying the asymmetric Sprague-Dawley airway morphometry [40, 41] and the clearance mode (https://www.ara.com/products/multiple-path-particle-dosimetry-model-mppd-v-304). The physiological parameters used were functional residual capacity (FRC), upper respiratory tract volume (URT), tidal volume, breathing frequency. For all these parameters, the default MPPD values for a given rat weight (which may differ from one study to another) were taken [42]. Specific exposure (and post-exposure) times were entered, whereas default rat clearance settings were used to estimate retention (mainly alveolar).

MMAD and its associated GSD were considered more relevant than CMD for mass-based dosimetry calculations [43].

When estimating fractions of MWCNT deposited (and especially the pulmonary fraction for this study), aspect ratio values are very important. However, these values are difficult to determine since they depend on the tendency of the MWCNTs to form agglomerates [14, 44]. For ‘fibre-like’ MWCNT such as MWNT-7 [45], the mean aspect ratio of individual fibres was used for MPPD modelling. However, for more entangled and ‘broadly-spherical’ MWCNT aerosols, the mean aspect ratios of the aerosols (estimated from transmission electron microscopy images of the aerosols collected on grids) rather than that of the original CNTs was used for dosimetry modelling (for NM403, Baytubes or Graphistrength for example) [14, 46, 47]. For aerosols containing both isolated CNTs and relatively spherical agglomerates (the case of NM401), an average value (=30 for NM401) between the aspect ratio of the original CNTs (=4/0.067 = 60) and that of a spherical particle (=1) was taken as default [14]. Any interpretation of the data relying on modelled retained doses must be considered with the significant uncertainties resulting from these choices in mind.

### Model fitting

Whole data given in the tables are expressed as the mean ± standard deviation. Dose-response curves (%neutrophils as a function of retained NM surface area per lung weight) were fitted to a sigmoidal curve, based on average values rather than individual animal data using the Hill equation (general equation for a sigmoidal dose-response curve) with the following form [48]:

$$ \% neutrophils=\%{neutro}_{in\ controls}+\frac{\%{neutro}_{max}-\%{neutro}_{in\ controls}}{1+{\left(\frac{EC50}{deposited\ surface\ area}\right)}^{Hill\ slope}} $$

where:

- *%neutro*_*in controls*_ (= 1.8%) corresponds to the average basal %neutrophils (endogenous) measured in all the (air exposed) control groups regardless of post-exposure time and rat strain, in this work and in previous studies from our laboratory [14, 35],
- *%neutro*_*max*_ is the asymptotic maximum response observed only for high enough NM doses (typically between 70 and 80%); by default, this value was set to 75% for modelling,
- *EC50* is the retained surface area dose (per lung weight) that provokes a response halfway between baseline (*%neutro*_*in controls*_) and the asymptotic high maximum response (*%neutro*_*max*_),
- *Hill slope* quantifies the steepness of the dose-response curve at EC50.

Both EC50 and Hill slope were determined by fitting a curve to the specific dataset under consideration using Statgraphics Centurion XVIII Software (Version 18.1.06) (StatPoint Technologies, Inc., Warrenton, VA, USA). The 95% confidence limits were established from the asymptotic standard errors.

## Results

### Aerosol monitoring and characterization

Table 1 summarizes the target and actual mass concentrations delivered as well as the main characteristics of the three benchmark NM aerosols: number concentration, count modal aerodynamic diameter (CMoAD) and associated GSD, MMAD, and aerosol effective density (average aerosol mass per volume based on mobility diameter [49]). Representative transmission electron microscopy images of the aerosols and their corresponding particle number (or mass) size distributions are provided in Supplemental 3 and 4, respectively.

**Table 1:** Main characteristics of TiO_2_ P25, Printex-90, and MWNT-7 aerosols produced for inhalation studies

Material	Target concentration (mg/m^3^)	Actual concentration (mg/m^3^)	Number concentration^c^ (particle×10^4^/cm^3^)	MMAD^d^ (μm)	CMoAD^e^ (μm)	GSD^e^	Aerosol effective density (g/cm^3^)^f^
P25	15 15 *(2-week)* 5^a^ 5^a^ *(2-week)* 1.5^a^ 1.5^a^ *(2-week)*	15.3 ± 3.98 15.3 ± 3.54 5.02 ± 0.39 5.04 ± 1.30 1.59 ± 0.45 2.20 ± 0.64	5.1 ± 1.7	1.56	0.31	1.72	1.70
P25	5^b^	5.09 ± 0.65	27 ± 2	0.40	0.17	1.82	0.90
Printex-90	50 15^a^ 5^a^	50.1 ± 3.89 15.0 ± 1.24 4.89 ± 0.39	35 ± 14	0.94	0.03 & 0.20	1.97 & 2.11	0.35
MWNT-7	1.5 0.5^a^ 0.15^a^	1.69 ± 0.49 0.47 ± 0.15 0.13 ± 0.02	0.14 ± 0.05	1.78	0.40	1.69	0.45

^a^6 h-equivalent concentration created by modulating the time for which animals were exposed to the aerosols produced from a dry powder generator

^b^aerosol produced from a nebulized suspension

^c^Measured by CPC particle diameter d_p_ < 3 μm

^d^Determined from cascade impactor (DPLI+) sampling, subsequent gravimetric analysis and further data inversion to account for particle deposition probabilities [50]

^e^Determined from a log-normal fitting of the number size distribution provided either by SMPS or APS measurements

^f^Aerosol effective densities were estimated by merging SMPS and APS number size distributions and assuming spherical particles [51]. These data shall be considered as indicative values

The mean actual NM aerosol concentrations never deviated by more than 13% from the target concentrations; with most deviations at less than 3%. As specified in the OECD TG 412 guideline [30], the test substance concentration sampled in the animals’ breathing zone in an inhalation chamber should not deviate from the mean chamber concentration by more than ±20% for solid aerosols. Variations of 25 to 30% were observed under certain conditions, when the generation capacities were pushed to the limits [P25 by rotating brush generator (RBG) at 15 mg/m^3^ or MWNT-7 by acoustic generator at 1.5 mg/m^3^]. To ensure sufficient exposure of the lower respiratory tract (alveolar region) in rats, the aerosols met the following standard: MMAD ≤2 μm with GSD between 1 and 3.

Despite similar CMoAD (0.17 instead of 0.31 μm), TiO_2_ aerosols produced by nebulization had a 4-fold smaller MMAD (0.4 μm) than when produced by RBG (1.56 μm) (Supplemental 4). When normalized to mass concentration, the number concentration for the aerosol produced by nebulization was around 16-fold higher (5.4 × 10^4^ particles/cm^3^ per mg/m^3^) than that obtained with a RBG (3.4 × 10^3^ particles/cm^3^ per mg/m^3^). The particle size distribution in number for the Printex-90 aerosol was bimodal (CMoADs of 0.03 and 0.2 μm), its total mass-normalized number concentration (7.0 × 10^3^ particles/cm^3^ per mg/m^3^) was of the same order of magnitude (~ 2-fold higher) as that of TiO_2_ RBG aerosol, but nearly 8-fold higher than that for MWNT-7 (9 × 10^2^ particles/cm^3^ per mg/m^3^).

### Lung burden & neutrophil influx following exposure to the three benchmark NMs

As expected, exposure to increasing aerosol concentrations induced increasing lung burdens (deposition). Over time, a fraction of the deposited particles was cleared from the lung and the amount retained decreased (Table 2). Details of the cytology results as well as body and lung weights are available in Supplemental 5.

**Table 2:** Neutrophil influx (normalized relative to total cell count in BALF) and retained NM mass and surface area lung burden observed at various post-exposure times after 4 weeks’ (or as indicated) nose-only inhalation exposure in rats

Material	Target concentration (mg/m^3^)	Post-exposure time	n =	Retained amount measured (mg / g lung)	Retained surface (cm^2^ / g lung)	Neutrophils (%)^*c*^
P25	15	D3 W4 W26	6 6 6	3.809 ± 0.641 3.272 ± 1.144 1.328 ± 0.873	2095 ± 327 1800 ± 629 730 ± 480	40.9 ± 7.0 57.7 ± 8.8 19.9 ± 12.7
	15 (2-week)	D3	10	1.699 ± 0.241	935 ± 123	22.0 ± 8.7
	5^a^	D3 W4 W26	6 6 6	1.368 ± 0.177 1.386 ± 0.384 0.756 ± 0.429	752 ± 90 762 ± 211 416 ± 236	13.5 ± 4.9 12.4 ± 7.5 1.7 ± 1.3
	5^a^ (2-week)	D3	10	0.672 ± 0.127	370 ± 65	5.1 ± 4.0
	1.5^a^	D3 W4 W26	6 6 6	0.511 ± 0.125 0.296 ± 0.041 0.097 ± 0.074	281 ± 64 163 ± 23 54 ± 41	2.7 ± 2.9 2.5 ± 0.6 2.2 ± 2.6
	1.5^a^ (2-week)	D3	10	0.246 ± 0.060	135 ± 31	2.3 ± 2.6
P25	5^b^	D0 D3 W4 W13 W26	6 6 6 6 6	1.372 ± 0.105 1.286 ± 0.152 0.585 ± 0.055 0.258 ± 0.036 0.122 ± 0.008	700 ± 54 656 ± 78 298 ± 28 132 ± 18 62 ± 4	4.4 ± 0.9 8.1 ± 2.4 1.2 ± 0.5 1.0 ± 0.6 1.8 ± 0.7
Printex-90	50	D3 W4 W26	6 6 6	2.467 ± 0.131 2.950 ± 0.492 3.708 ± 0.631	7797 ± 414 9322 ± 1555 11,717 ± 1994	72.9 ± 5.6 52.3 ± 7.4 48.3 ± 6.4
	15^a^	D3 W4 W26	6 6 6	1.340 ± 0.269 1.418 ± 0.234 1.080 ± 0.177	4234 ± 850 4481 ± 739 3413 ± 559	55.1 ± 19.0 41.0 ± 10.0 9.5 ± 6.0
	5^a^	D3 W4 W26	6 6 6	0.895 ± 0.228 1.428 ± 0.372 0.553 ± 0.161	2827 ± 721 4512 ± 1176 1747 ± 509	18.8 ± 4.2 5.2 ± 1.8 2.2 ± 2.3
MWNT-7	1.5	D3 W4 W26	6 6 6	0.834 ± 0.460 0.310 ± 0.181 < 0.060	192 ± 106 71 ± 42 < 14	44.7 ± 9.9 28.3 ± 8.9 10.1 ± 5.3
	0.5^a^	D3 W4 W26	6 6 6	0.397 ± 0.217 < 0.060 < 0.060	91 ± 50 < 14 < 14	30.6 ± 13.5 9.0 ± 4.4 8.3 ± 8.6
	0.15^a^	D3 W4 W26	6 6 6	< 0.060 < 0.060 < 0.060	< 14 < 14 < 14	10.2 ± 8.1 4.9 ± 6.5 4.7 ± 5.3

^a^6 h-equivalent concentration created by modulating the exposure time to the aerosols

^b^aerosol produced from a suspension by nebulization

^**c**^For each experiment, control groups (exposed to filtered air) were monitored in parallel to groups of aerosol-exposed animals. In control animals, levels of % neutrophils never exceeded 3.6% (1.8% on average) regardless of the post-exposure time

Following exposure to P25 agglomerated aerosols generated from dry powder, the amount of TiO_2_ retained within the lung (normalized to the P25 airborne concentration in mg/m^3^) was around 274 μg/g lung (per mg/m^3^) (Supplemental 6). This was 25% higher than the amount of aerosol (less agglomerated) retained following exposure to the nebulized P25 aqueous suspension (221 μg/g lung per mg/m^3^). Considering the actual respiratory parameters of rats (i.e., tidal volume, breathing frequency and minute ventilation measured by thoracic plethysmography during exposure), the fractions retained were quite similar: 13.3 and 15.1% of the P25 aerosol dose was inhaled. The fractions retained (sum of pulmonary and tracheobronchial fractions) were somewhat higher than those estimated by the MPPD model: 8.2 and 10.1%, respectively. Using first order kinetic models, an elimination half-time of 52 days was estimated for the nebulized (5 mg/m^3^) P25 aerosol, whereas it was 70 days for the 1.5 mg/m^3^ and exceeded 98 days for the 5 and 15 mg/m^3^ (dry) aerosols (Supplemental 6).

Printex-90 exposures did not deliver the same normalized deposited dose (normalized to Printex-90 airborne concentration) at D3; the dose decreased with increasing airborne concentration (from 185 to 51 μg/g lung per mg/m^3^ for 5 and 50 mg/m^3^ exposures, respectively) (Supplemental 7). Very little elimination of particles over time was recorded; a tendency to increase was even sometimes observed (perhaps due to redistribution of particles in the right median lobe?). Whatever the case, the elimination half-time was greater than 180 days.

For MWNT-7, the elimination half-time was less than 28 days, although the precision of this value is reduced due to the large number of dose measurements below the limit of quantification (Table 2).

Except for exposure to P25 at 1.5 mg/m^3^, a significant dose dependent influx of neutrophils was observed shortly after the end of the exposure for each type of aerosol (on day 3 post-exposure). The highest neutrophil influx levels (> 40%) were observed following exposure to P25 at 15 mg/m^3^, to Printex-90 at 50 and 15 mg/m^3^, and to MWNT-7 at 1.5 mg/m^3^. The magnitude of the influx declined over time but remained above that of control animals in the majority of cases at W26 (180 days post-exposure.).

### Selection of studies from the literature for comparison

Table 3 presents the details of the publications identified reporting on studies involving TiO_2_, MWCNT, and CB inhalation, and meeting our selection criteria. For comparison, the information from the aforementioned exposure is also included in Table 3.

**Table 3:** Overview of key parameters of inhalation studies included in the analysis presented

Material	Name	Primary particle diameter (nm)	BET surface area (m^2^/g)	Aerosol characteristics MMAD (μm) (GSD)	Type of exposure^a^ & duration	Mass concentrations (mg/m^3^)	Post-exposure time^b^	Lung retained dose	Rat Sex and strain	Age (or weight) at start of exposure	Reference(s)
**TiO**_**2**_	P25	21	51	*1.56 (1.72)*	*[NO]* *6 h/d 5 d/w* *for 2 or 4 w*	*1.5*^*c*^*, 5.0*^*c*^*, 15*	*D3, W4, W26*	*Measured*	*Female Sprague-Dawley*	*13-week*	*This work*
				*0.40 (1.82)*	*[NO]* *6 h/d 5 d/w* *for 4 w*	5	*D0, D3, W4, W13, W26*	*Measured*	*Female Sprague-Dawley*	*13-week*	*This work*
				0.91 (2.19)	[NO] 6 h/d 5 d/w for 4 w	10	D0, D3, W4, W13, W26	Measured	Male F344	13-week	Chézeau, et al. [35] Gate, et al. [36]
				0.91 (2.19)	[NO] 6 h/d 5 d/w for 4 w	10	D0, D3, W4	Measured	Male F344	19-month	Gate, et al. [36] Valentino, et al. [52]
				1.44 ± 0.57 (2.60 ± 0.38)	[WB] 6 h/d 5 d/w for 13 w	10	D0, W4, W13, W26, W52	Measured	Female F344	7-week	Bermudez, et al. [53]
				0.7–1.1 (2.3–3.4)	[HO] 6 h/d for 5 d	2, 10, 50	D0, D16	Measured	Male Wistar	9-week	Ma-Hock, et al. [54]
	Fine (anatase)	2100 ±1500	8.8	1.0 (2.6)	[WB] 6 h/d for 5 d	50	D7, D14, W4, W9	Measured	Male F344	180–200 g	Driscoll, et al. [55], Driscoll, et al. [56]
				1.2–1.3 (1.9–2.6)	[NO] 6 h/d 5 d/w for 4 w	0.1, 1, 10	D7	Measured	Female F344	11–13 weeks	Henderson, et al. [57]
	Rutile pigment		6	1.44 ± 0.09 (1.71 ± 0.23)	[WB] 6 h/d 5 d/w for 13 w	10, 50, 250	D0, W4, W13, W26, W52	Measured	Female F344	7-week	Bermudez, et al. [58]
	Bayertitan T (rutile)	1800	1.9	1.1 (1.6)	[NO] 6 h/d 5 d/w for 13 w	5	D1, W13	Measured	Male & female Wistar	8-week	Morimoto Y., et al. [59]
**CB**	Printex-90	14	300	*0.94 (2.11)*	*[NO]* *6 h/d 5 d/w for 4 w*	*5*^*c*^*, 15*^*c*^*, 50*	*D3, W4, W26*	*Measured*	*Female Sprague-Dawley*	*13-week*	*This work*
				1.4–1.6 (2.4–2.7)	[WB] 6 h/d 5 d/w for 13 w	1.1, 7.6, 50.3	D0, W13, W44	Measured	Female F344	7-week	Elder, et al. [60] Gallagher, et al. [61]
				0.3–0.5 (2.0–3.2)	[NO] 6 h/d 5 d/w for 4 w	2.9, 9.9, 32.9	D0	Measured	Male Sprague-Dawley^d^	6-week	Lim, et al. [62]
	Elftex 12	37	43	2.02–2.42 (1.76–1.90)	[WB] 7 h/d 5 d/w for 12 w or 16 h/d 7 d/w or 6 h/d 5 d/w or 4 h/d 1 d/w for 6 or 12 w	3.5, 13.2, 100	D1 or D0, W12, W24	Measured	Female F344	11–15 weeks	Wolff, et al. [63] Henderson, et al. [64]
	Monarch 880	16	220	0.88 (3.3)	[WB] 6 h/d 5 d/w for 6.5 w or 13 w	1.1, 7.1, 52.8	D0, W12, W32	Measured	Male F344	200–250 g	Driscoll, et al. [65]
	Sterling V	70	37	0.8 (3.2)	[WB] 6 h/d 5 d/w for 13 w	48.2	D0, W13, W48	Measured	Female F344	7-week	Elder, et al. [60] Gallagher, et al. [61]
**MWCNT**	MWNT-7	∅ 88 ± 5 L 5.0 ± 4.5	24–28	1.78 (1.69)	*[NO]* *6 h/d 5 d/w* *for 4 w*	0.15^c^, 0.5^c^, 1.5	*D3, W4, W26*	*Measured*	*Female Sprague-Dawley*	*13-week*	*This work*
				1.2–1.3 (2.4–3.4)	[WB] 6 h/d 5 d/w for 2 w	0.2, 1, 5	D0	Computed	Male & female F344	6-week	Umeda, et al. [66]
				1.4–1.6 (2.3–3.0)	[WB] 6 h/d 5 d/w for 13 w	0.2, 1, 5	D0	Measured	Male & female F344	6-week	Kasai, et al. [45]
	Baytube (micronized)	∅ 10–15 L 0.30 (0.05–1.35)	210	2.9 (1.9)	[NO] 6 h	11, 241	D7, W13	Measured	Male Wistar	9-week	Ellinger-Ziegelbauer, et al. [67] Pauluhn, et al. [68]
			255	3.05 (2.0)	[NO] 6 h/d 5 d/w for 13 w	0.1, 0.4, 1.5, 6	D1, W4, W13, W26	Measured	Male Wistar	9-week	Pauluhn [46], Pauluhn, et al. [68]
	Nikkiso	∅ 44 L Not provided	69	∅: 63 nm (1.5)^e^ L: 1.1 mm (2.7)^e^	[WB] 6 h/d 5 d/w for 4 w	0.37	D3, W4, W13	Computed	Male Wistar	9-week	Morimoto Yasuo, et al. [69] Oyabu, et al. [70]
	NM-401	∅ 67 (24–138) L 4.0 ± 0.37	18	0.79 (1.83)	[NO] 6 h/d 5 d/w for 4 w	0.54, 1.49	D3, W4	Measured	Female Sprague-Dawley	13-week	Gate, et al. [14]
	Graphistrength C100 (NM-402)	∅ 12.1 L 1.07 ± 1.10	225.6	1.62–2.03 (2.5–4.7)	[NO] 6 h/d 5 d/w for 13 w	0.05, 0.25, 5	D1, W13	Computed	Male & female Wistar	8-week	Pothmann, et al. [47]
				0.7–2.0 (2.1–4.1)	[HO] 6 h/d 5 d/w	0.15, 0.57, 2.86	D3, D24	Computed	Male & female Wistar	8–9 weeks	Ma-Hock, et al. [71]
	NM-403	∅ 12 (5–37) L 0.40 ± 0.03	135	1.94 (1.48)	[NO] 6 h/d 5 d/w for 4 w	0.50, 1.48	D3, W4	Computed	Female Sprague-Dawley	13-week	Gate, et al. [14]
	JC 162 Incheon	∅ 8–10 L 0.1–0.2	400–600	0.38–1.02 (2.34–3.08)	[NO] 6 h/d 5 d/w for 4 w	0.26, 1.44, 4.25	D1, D7, W4	Measured	Male Sprague-Dawley	8-week	Kim JK, et al. [72]
	Nanocyl NC 7000	∅ 5–15 L 0.1–10	250–300	0.7–2.0 (2.1–4.1)	[HO] 6 h/d 5 d	2.4, 8.4, 29.8	D3, D23	Computed	Male Wistar	9-week	Ma-Hock, et al. [73]

^a^WB: whole-body; HO: head-only; NO: nose-only

^b^D for day, W for week

^c^6h-equivalent concentrations created by modulating the exposure time to the aerosols

^d^normal and overweight rats

^e^Geometric mean (geometric standard deviation) measured by scanning electron microscopy

The results of the various studies were analysed based on information relating to the physiochemical properties of the material (primary particle dimensions, mass-specific BET surface area), the corresponding aerosol size distribution (MMAD, GSD), the rats used (sex, strain, age), the exposure details (type, duration, mass concentration) and the NM lung burden retained (NM mass per lung weight) at specified post-exposure times. Only data sets which provided all the parameters mentioned are listed in Table 3; post-exposure times for which the cytology results could not be linked to lung burden (or vice versa) or for which the lung burden could not be estimated from the aerosol parameters using dosimetric modelling (MPPD) were not considered.

Overall, three strains of rats were used in the different studies: Fisher 344 (F344), Sprague-Dawley, and Wistar. Female or male animals were used; some studies investigated both sexes and reported no notable sex-related differences [45, 47, 59, 66]. The age at the beginning of inhalation exposure generally ranged from 6 to 13 weeks, but 19-month-old (elderly) rats were used in one study [52]. There was no preferred mode of exposure (NO, HO, WB). Exposure durations varied from 1 day to 13 weeks, and post-exposure monitoring times also varied considerably, ranging from D0 (immediately following exposure) up to W52 (1 year of recovery).

Aerosol were mainly generated by dry methods, with aerosols produced from (dry) powders which were aerosolized by a combination of mechanical forces and air carriers. Several systems were used: Wright dust feeder [60, 63, 67], brush generators [54, 58], jet mill [55], Venturi jet [57, 65], acoustic generator [72], dedicated home-made dust feeder [74], etc. Sometimes, powders were milled before aerosolization [47, 67]. Only two reports described wet-based methods involving nebulization of suspensions of NMs (CB [62] or MWCNT [69]) in distilled water (possibly with Triton [56]).

For TiO_2_, two studies [53, 54] on P25 were considered along with two previous studies from our laboratory [35, 36, 52]. Except for P25, no other inhalation study on nano-TiO_2_ was selected as they are rare. Data for three distinct fine TiO_2_ (anatase and rutile) preparations were available [55–59].

Among the CB inhalation studies, only six met the selection criteria for integration into this analysis, all dealt with furnace CB. In addition to two studies on Printex-90 [60–62], four others investigated Elftex 12, Monarch 88, and Sterling V [60, 61, 63–65].

The CNT inhalation studies best met the imposed criteria, because the authors provided more experimental details. Thus, investigations of eight different CNTs meeting our selection criteria were identified. Among these studies, information was available on Baytubes [46, 67, 68], JC 162 Incheon [72], Nanocyl NC7000 [73], Nikkiso [69, 70], NM401 [14], NM402 (Graphistrength C100) [47, 71] and NM403 [14], as well as MWNT-7 [45, 66]. These CNTs were not functionalized and differed in diameter and length.

It should be noted that the aerosol characteristics reported in studies of P25, Printex-90, or MWNT-7 tended to differ considerably. Thus, the aerosol mass concentrations reported spanned a wide range (≥ 50-fold) for CB (1 to 50 mg/m^3^) and CNT (0.1 to 6 mg/m^3^), and 2500-fold differences were even noted for TiO_2_ (between 0.1 and 250 mg/m^3^).

### Relationships between neutrophil influx and retained surface area dose

**Fig. 1:**
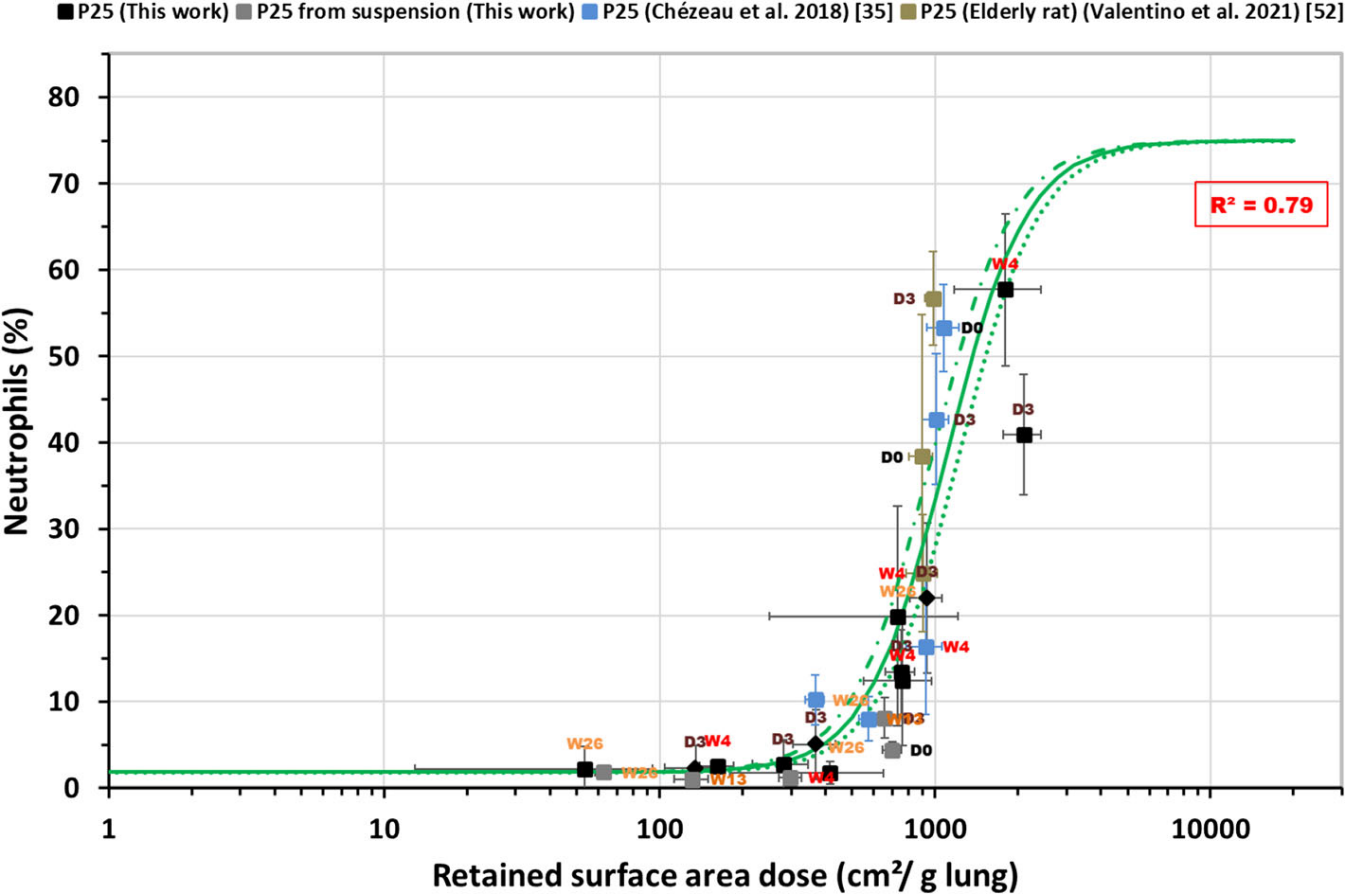
Neutrophil influx observed at various post-exposure times (D0 to W26) following 4-week (square) or 2-week (diamond) inhalation exposures to TiO_2_ P25 depending on the measured retained surface area dose measured (see Table 3). The solid green line shows the fit of the regression model. The dotted green lines delimit the 95% confidence interval of the regression model

Figure 1 shows the neutrophil influx into the lung observed at various post-exposure times following 4 weeks’ inhalation exposure (or on D3 following a 2-week exposure) to TiO_2_ P25. Influx is represented as a function of the retained surface area dose normalized relative to lung weight. Further details can be found in Tables 2 and 3. The entire dataset was well described by a sigmoid curve (*R*^*2*^ = 0.79; fitting parameters are provided in Table 4) with an onset dose – leading to 6% neutrophil influx (defined as 3 × SD _%neutro in controls_) – of 430 cm^2^/g lung that is not influenced by post-exposure time.

**Table 4:** Exposure conditions, curves fitting parameters and onset surface area doses leading to 6% neutrophil infiltration for the different classes of NM studied

Material	Exposure	Reference	Number of conditions^a^	Hill equation parameters^b^			Onset surface area dose (cm^2^/g lung) for 6% neutrophils^c^
				EC50^d^	Hill slope^d^	R^2^	
P25	2- & 4-week	This work + [35, 36, 52]	25	1097 [973–1220]	2.98 [1.89–4.09]	0.79	430
P25	13-week	[53]	5	4683 [4109–5256]	5.86 [0.98–10.7]	0.91	2904
P25	1-week	[54, 55]	7	418 [285–552]	2.88 [0.89–4.87]	0.93	158
Fine TiO_2_	All duration	[54–59]	25	2069 [1990–2148]	7.34 [5.36–9.32]	0.99	1413
Printex-90	4-week	This work	9	5262 [2795–7728]	2.22 [−0.34–4.78]	0.55	1492
Printex-90	13-week	[60, 61]	9	10,038 [6348–13,728]	1.37 [0.50–2.24]	0.88	1301
MWNT-7	2-, 4- & 13-week	This work + [45, 66]	22	61.6 [47.6–75.5]	1.08 [0.82–1.34]	0.81	4.6
+ NM401, NM402, NM403, Nanocyl NC7000	All duration	+ [14, 47, 71, 73]	+ 32	90.1 [58.9–121.4]	0.85 [0.62–1.09]	0.77	3.3
Micronized Baytubes	13-week		16	6366 [2407–10,326]	0.72 [0.49–0.95]	0.87	130

^a^considered for modelling; conditions means the combinaison of experimental conditions (aerosol, concentration, post-exposure time point, …)

^b^low and high asymptotic %neutrophils values are “in controls” = 1.8% and “max” = 75%, respectively (see § Model fitting)

^c^6 = 3 × SD(%neutro _in controls_)

^d^The values in brackets define the asymptotic 95% confidence intervals

**Fig. 2:**
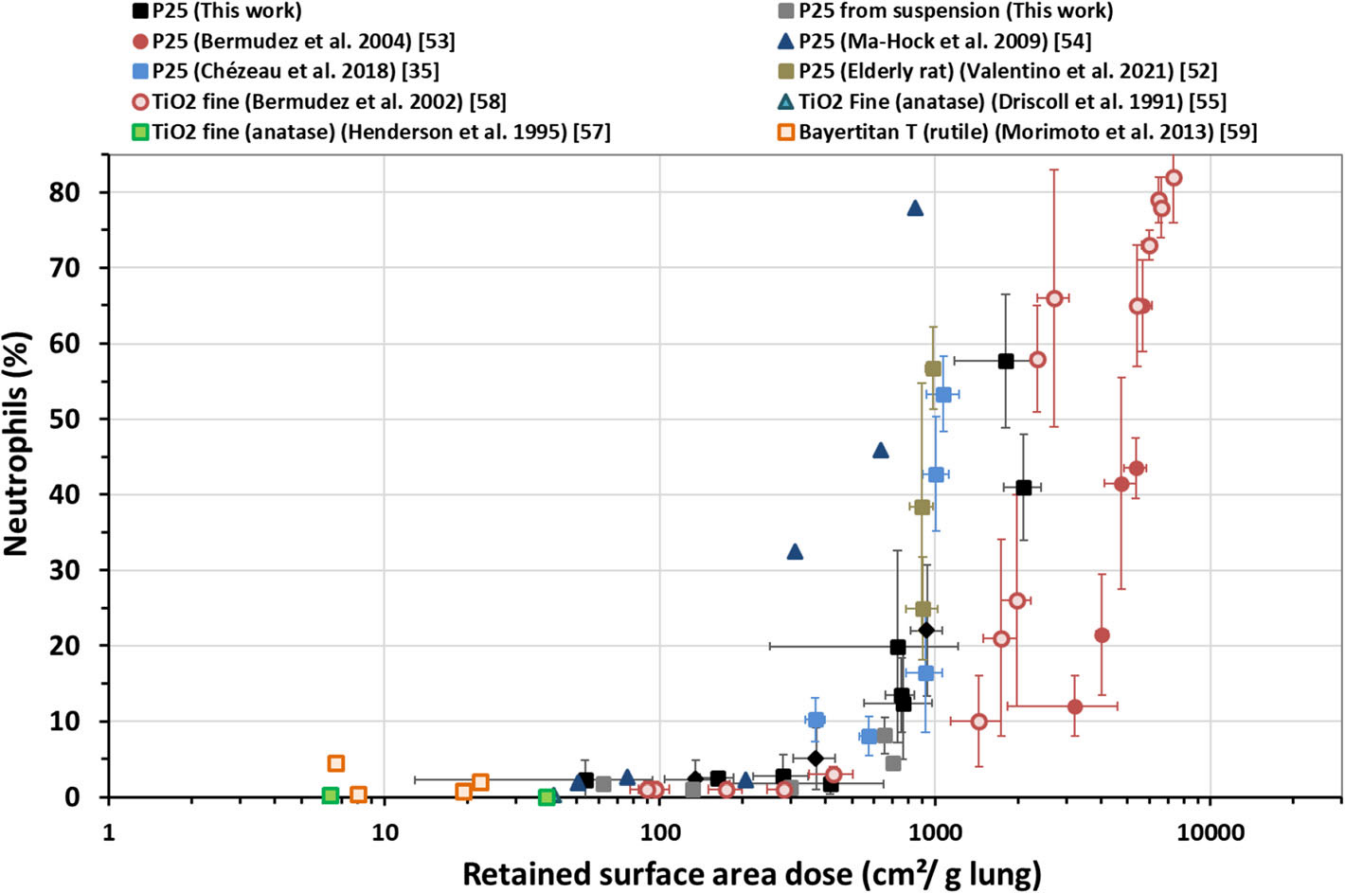
TiO_2_ material-induced effect on neutrophil influx depending on retained surface area dose measured for various exposure times: 1-week (triangle), 2-week (diamond), 4-week (square) or 13-week (circle) (see Table 3). Both nanoparticles (unicolor) and fine particles (bicolour) were considered. Details of the post-exposure times are provided in Supplemental 8

**Fig. 3:**
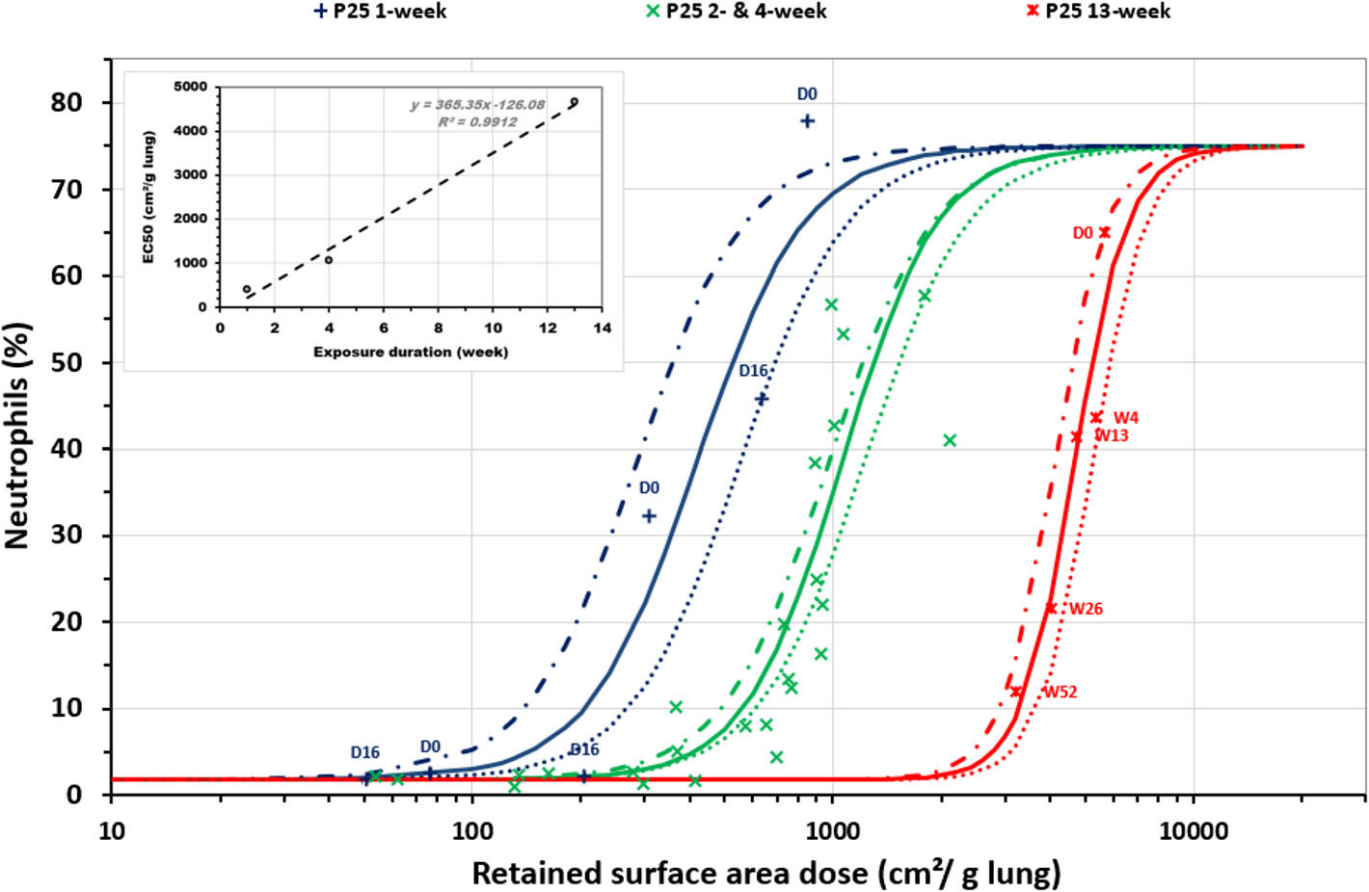
Neutrophil influx depending on P25-retained surface area dose: dose rate effect. 1-week (blue +), 2- and 4-week (green ×), and 13-week (red sign). The blue, green and red dotted lines represent the fit of the regression models for the 1-week, 2- and 4-week, and 13-week exposures, respectively. The dotted blue, green and red lines delimit the 95% confidence intervals of the regression models

Considering TiO_2_ exposures (P25 or other types of TiO_2_ (fine rutile or anatase)) with different exposure durations and post-exposure times (immediately following exposure (D0) up to a year post-exposure (W52) (Supplemental 8)), short-term exposure appeared to be more inflammogenic than longer-term exposure (Fig. 2). Indeed, distinct sigmoidal fits were obtained for curves corresponding to 1-week (acute), 4-week (+ 2 week) (subacute) and 13-week (subchronic) exposure to P25 (Table 4). Based on the 95% confidence intervals around these three sigmoid curves, the differences were statistically significant (Fig. 3). The onset doses whatever the post-exposure time assessed – for 1-week, 4-week and 13-week exposure were 160, 430 and 2900 cm^2^/g lung, respectively (Table 4). This onset dose, or even better the EC50 (Table 4), was proportional to the duration of exposure (upper left corner of Fig. 3). It should be noted that all the data considered in this analysis were in fact produced by only three teams (including ours), which only considered one duration of exposure. Therefore, it cannot be excluded that part of the significant difference could have its origin in this fact. Due to the low BET surface area of fine rutile and anatase TiO_2_, the sigmoid curve fit reported for these particles is mainly linked to the data for 13-week exposure to rutile reported by Bermudez, et al. [58] with high aerosol mass concentrations (10, 50 and 250 mg/m^3^) (see Table 3). This curve was nevertheless statistically distinct from those obtained for 4-week and 13-week P25 exposures (see the EC50 confidence intervals on Table 4).

**Fig. 4:**
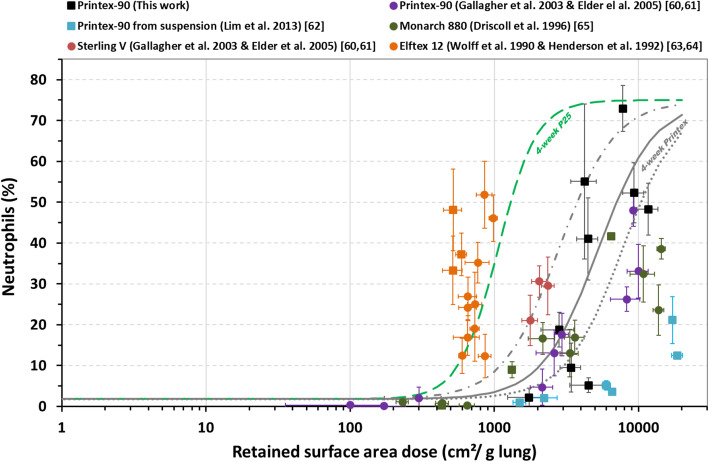
CB-induced effect on neutrophil influx depending on the retained surface area dose measured following 4-week or 6-week (square) and 13-week (circle) inhalation exposure. Details of the post-exposure times are provided in Supplemental 9. The grey line shows the fit of the regression model for 4-week Printex-90 exposure (from this work). The dotted grey lines delimit the 95% confidence interval of the regression model

For Printex-90 (4-week exposure and recovery time ranging from D3 to W26) the onset dose was around 1490 cm^2^/g lung based on the sigmoidal curve fitted to the data reported here (*R*^*2*^ = 0.55) (Fig. 4, Table 4, and Supplemental 9). This curve fitting did not allow us to describe the 4-week nebulized Printex data (notable by the very weak inflammatory response induced; onset ~ 8000 cm^2^/g lung) [62]. However, these data were statistically similar to the 13-week Printex-90 data [60, 61], with only a slight shift towards a higher retained dose and no effect on onset dose (~ 1300 cm^2^/g lung, *R*^*2*^ = 0.71) (Table 4). Unlike exposure to Sterling V [60, 61] and Monarch 880 [65], which produced dose-response curves close to those of Printex-90, the curve for Elftex [63, 64] was closer to the 4-week P25 curve (Fig. 4). It should be noted that the points (orange or green squares) representing the results for the 6- (or 6.5-) week exposures to Elftex-12 (or Monarch 880) tended to lie the left (i.e., lower retained surface dose) of the groups of points representing the 13-week exposures. The limited data available did not provide sufficient statistical power to conclude on an effect of exposure duration.

**Fig. 5:**
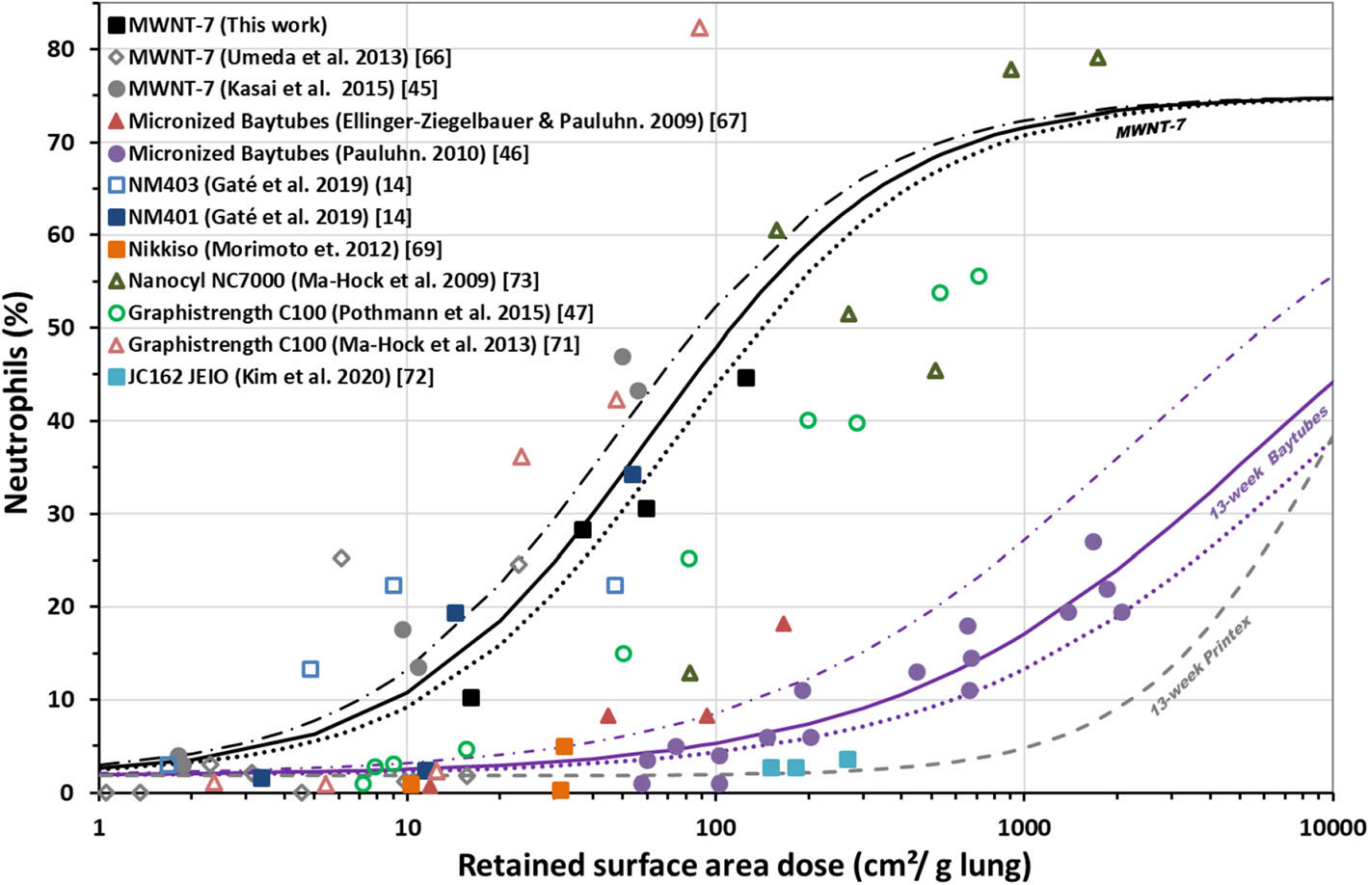
MWCNT-induced effect on neutrophil influx depends on the retained surface area dose (measured or estimated) following 1-week (triangle), 2-week (diamond), 4-week (square) or 13-week (circle) inhalation exposure. Details of the post-exposure times are provided in Supplemental 10. Open symbols indicate that the retained surface area dose was estimated using the MPPD model. The retained surface “carbon” dose applied for micronized Baytubes was calculated based on Co analysis (0.53% w/w in pristine MWCNT). The black and purples lines show the regression model fits for MWNT-7 exposure (all durations) and 13-week exposure to micronized Baytubes, respectively. The dotted black and purples lines delimit the 95% confidence intervals of the corresponding regression models

Regarding CNTs (Fig. 5 and Table 4), we observed a good correlation between %neutrophils and the retained surface area dose (*R*^*2*^ = 0.81) for MWNT-7 data from 2-, 4- and 13-week exposures (reported here or in [45, 66]) regardless of the post-exposure times (Supplemental 10). The MWNT-7 dose-response relationship was very consistent with that obtained by including data from NM-401, NM-402 (Graphistrength C100), NM-403, and Nanocyl NC7000, especially if we consider that not all data were obtained with the same exposure duration (*R*^*2*^ = 0.77) [14, 47, 71, 73]. For these five types of MWCNT, the threshold concentration triggering neutrophil influx was very low (6% influx triggered by between 3 and 5 cm^2^/g lung) (Table 4). In contrast, micronized Baytubes [46, 67, 68] and JC162 [72] were much less inflammogenic. Exposure to micronized Baytubes for 13 weeks followed a dose-response curve very similar to that established for 13-week Printex-90 exposure. Nikkiso MWCNT also seemed to relate to this second “family” of CNTs, although the small number of data points available makes this conclusion difficult to affirm [69, 70].

## Discussion

The aim of this study was to determine if retained surface area in the lung was a reliable metric to determine the inflammogenic potential of different classes of NM.

Inflammation is a complex process at the molecular level in an individual cell but also in the communication between different cell types. However, for the purposes of this quantitative multi-study analysis we had to select a widely used indicator of inflammation namely the “neutrophil influx” expressed by %PMN.

It is clear that the toxicological results obtained by inhalation must be interpreted in the light of deposited (retained) doses (whether it is mass or surface) and not based on inhaled aerosol concentrations since particle deposition is significantly influenced by the characteristics of the aerosols inhaled. In addition, this internal deposited dose is essential to translate toxicological dose-response data into risk assessment and exposure limits [11].

Deposited dose expressed as mass has been the most used metric to date. This metric is generally simple to monitor and does not change over time. However, although it can be useful when studying dose-effect relationships for a specific material, it is less relevant when considering different materials, and even less so when the materials are from different families. Surface area is a more relevant dose metric, in particular for hazard grouping [12], as it can be used to identify (or demonstrate the absence of) differences in effect between distinct particles.

Shape is another important predictor. On the basis of deposited surface area, a majority of CNTs (among the non-functionalized MWCNT studied here) are more inflammogenic than TiO_2_, which themselves induced a stronger inflammatory response than CBs. Indeed, we demonstrated the existence of distinct thresholds triggering an inflammatory response for the different classes of NMs. Thus, onset surface area doses estimated (for 4-week exposures in Table 4) for TiO_2_ and CBs at 430 and 1500 cm^2^/g lung, respectively, were two to three orders of magnitude higher than those estimated for the more potent MWCNTs (~ 4 cm^2^/g lung). The apparent heterogeneity in the results obtained with CB is difficult to explain, particularly when considering the more inflammogenic Elftex 12 [63, 64]. Possible explanations include the presence of metal impurities and highly toxic organic compounds. In addition, it appears that Printex-90 aerosol generated from an aqueous suspension displays significantly reduced surface-specific inflammogenicity [62]. This effect could be the result of water-induced passivation, as suggested by the lack of difference in surface-specific inflammogenicity after direct pulmonary application (no aerosol) of six types of CB suspensions with very different organic carbon content [75].

It should be noted that for PSLT, the onset surface area doses reported are at levels where lung overload conditions (reduction of lung clearance) have already been reached [76]. This marks a clear difference with the data available from inhalation with CNTs because, except for subchronic inhalation of Baytubes at 1.5 and 6 mg/m^3^ [46], overload conditions were not reported in studies measuring MWCNT lung burden.

MWCNT could be subdivided into two groups based on the dose-response for retained surface area and inflammation. The more ‘potent’ group comprised NM-401, NM-402 (Graphistrength), NM-403, Nanocyl NC7000, and MWNT-7. The less potent group (micronized Baytubes, JC162) behaved more like TiO_2_ and CB. Based only on the inflammatory response results reported here, it is impossible to determine which group Nikkiso falls into. In addition, for Nikkiso, the technique used for CNT preparation (grinding of a solidified body of MWCNT kneaded with fructose before soaking, filtration and treatment with hydrogen peroxide to remove fructose) and nebulization (from an aqueous suspension with 0.5 mg/mL Triton X-100) [69] may affect its reactivity.

Up to now, MWNT-7 is the only MWCNT classified by IARC as 2b (possibly carcinogenic to humans) (our choice to use it as a benchmark material was therefore dictated by this classification more than by its physicochemical properties), other CNT are classified as 3 due to inadequate or limited evidence of carcinogenicity when the IARC assessment was performed [77]. The results presented here confirm (Fig. 5) that it does not seem to be toxicologically justified to group all carbon nanotubes into a single substance category [78], even if it could be argued from a safety point of view since there is no clear physicochemical property by which to predict group membership for a CNT. It should be noted that the search for the key parameter(s) driving CNT toxicity is also complicated by the fact that physicochemical properties provided by the suppliers are often imprecise or even incorrect [79]. Despite the inherent difficulties, it has been established that length is a major determinant of CNT toxicity; long MWCNT conform to the fibre paradigm, and may, like asbestos, cause frustrated phagocytosis [8, 80–82]. The diameter and consequently the aspect ratio and rigidity of CNTs also significantly contributes to their biological effects. Thus, the rigidity of CNTs correlates strongly with both acute and chronic inflammation and frustrated phagocytosis [15, 83–85].

These considerations explain why MWNT-7, which is the best-known example of long and rigid CNTs (L = 5 μm and ∅ = 88 nm), may cause considerable damage to the lungs following pulmonary exposure, and may explain why NM-401 (L = 4 μm and ∅ = 67 nm) or Nikkiso (∅ = 44 nm) could induce a similar pathological pattern [14, 45, 69, 77].

The other CNTs investigated have smaller and more similar diameters (∅ ~ 10 nm) but ca. 50-fold varying lengths, ranging from a few microns (5 and 1 μm for Nanocyl NC7000 and Graphistrength C100, respectively) down to 0.3–0.4 μm (for Baytubes and NM-403) and even 0.1–0.2 μm for JC-162. In the aerosol phase, these entangled nanotubes form spherical, ovoid or elongated micronic agglomerates (in the case of JC-162, the agglomerates can even take the form of a macro tube measuring several hundreds of microns long and a few microns wide [72]). These forms limit their respirability and deposition in the alveolar region of the lung. However, our previous results from a comparative analysis of the transcriptome in the whole lung and the proteome in the BALF of rats exposed to NM-401 and NM-403 indicated that the latter (a short and/or tangled CNT usually considered less harmful) could induce pathological effects in the lung by a pathway differing from that triggered by NM-401 [86]. Indeed, following inhalation exposure to NM-401, we identified more differentially expressed genes involved in the fibrotic process than after NM-403 inhalation exposure. In addition, omics data revealed specific pathways dysregulated in NM-401 samples (e.g. cell cycle, lysosome, oxidative stress defense) in comparison to NM-403 samples (e.g. cytosolic DNA-sensing pathway, metabolic pathways).

Apart from the lower aspect ratio (around 20–25), the difference in “behaviour” between Baytubes and JC-162 on the one hand, and Nanocyl NC7000, Graphistrength C100, and NM-403 on the other, is difficult to rationalize based on simple physical parameters. Although Nanocyl NC7000 and Graphistrength C100 have aspect ratios of almost 100, that of NM-403 (~ 30–35) is just slightly higher than that of Baytubes. For the latter, authors claimed that the micronisation process (by ball milling) had no effect on the assemblage structure [46]. Nevertheless, it could be hypothesized that this treatment has sufficiently modified the surface to reduce its reactivity (to a level close to that of CBs). Investigations with other short CNTs should be performed to verify whether an aspect ratio of less than 20 can be considered a “safe” aspect ratio.

The determination of the CNT lung burden (or that of CB, because the methodological difficulties of detecting carbon within a carbon-rich matrix such as lung tissue are similar) reported in the various studies involved a variety of methods - measurement of Co catalyst present in the CNT [67], thermal [39] or thermo-optical analysis [72], X-ray diffraction and elemental carbon analysis [70], HPLC analysis [45], light extinction [63], etc. - which were not always validated according the required standards. However, it is important to point out that the uncertainties in lung burden determined cannot explain the extent of the differences observed, of one or two orders of magnitude. Likewise, the difference is such that estimates made using the MPPD model for MWNT-7, NM-403, Graphistrength C100, or Nanocyl NC7000 exposures would not lead to these NMs being classed in the other CNT subgroup.

Another important point demonstrated by the results presented here is that, when we focus on a family of NMs, the % neutrophils is related to the surface area dose retained within the lung regardless of the post-exposure time considered. Indeed, in cases of overloading (for TiO_2_ and CBs) or in the presence of biopersistent NM (for some MWCNTs), the % neutrophils remained high; in all other cases, the decrease in % neutrophils was directly associated with the remaining surface area over time. The relation for this association takes the form of a sigmoid. In other words, the NMs are not cleared or passivated over time due to bioprocessing.

Based on surface area deposited, and at least for the inflammation phenomena considered in this article, small particles exert similar effects to larger ones, as illustrated by the inflammation results reported by Bermudez et al. [53, 58] following subchronic inhalation of fine and ultra-fine TiO_2_ (Fig. 2). Similarly, as clearly demonstrated here with P25 and to a lesser extent with Printex-90 and Graphistrength C100, distinct aerosols (with non-identical agglomeration states depending on the generation mode) produced from the same starting material induce equivalent inflammatory responses at the same surface area deposited dose. Only differences in lung clearance kinetics will cause the inflammation to decrease more quickly over time in one case rather than another.

There is evidence that the dose rate is a significant factor explaining differences in responses when comparing distinct modes of administration (instillation vs. inhalation) [87, 88]. In general, the higher the dose rate, the smaller the surface area dose needed (for a given substance) to trigger a specific inflammatory response. In addition, this dose rate effect appears all the more important for substances with a low inflammogenic potential. For example, for NM-401 and NM-403, instillation and inhalation for 4 weeks produced the same dose-response curve [14]. Considering only inhalation with different exposure durations, the analysis of published data alongside data produced by our laboratory revealed different onset doses following 1, 2 and 4, or 13 weeks’ exposure to P25 TiO_2_. Based on the datasets available (and the corresponding limited statistical power) we cannot draw a definitive conclusion on the dose rate effect for CB (or micronized Baytubes which behave like a CB). However, we can report trends for Printex-90, Monarch 880, and Elftex 12. The more inflammogenic MWCNT was not associated with an effect of exposure duration.

The significance/predictivity of the “retained surface area” dose for inflammogenic hazard ranking is quite striking. It nevertheless relies on many prerequisites or elements of information which are not always available in publications, demonstrated by the small number of studies suitable for inclusion in this work. The surface area calculation retained relied on lung burden measurements for inhalation, but lung burden is sometimes difficult to measure - particularly for CB and MWCNT - and no standard method has yet been developed [39, 44]. Alternatively, a well-conducted characterization of the aerosol could supplement this dosage part, and we really consider that efforts in this direction are worthwhile [34]. Indeed, the deposited (or retained) surface area could be estimated from powder S_BET_, airborne mass concentration, effective density, and regional deposited fractions using the aerosol’s particle size distribution and the MPPD model (with or without the clearance module) [40–42]. It should be noted that using the BET surface area to estimate deposited surface could still be challenging with porous particles due to their high surface area. Recent studies of solid and porous SiO_2_ particles suggest that the internal surface area contributes to inflammation at least to some degree [19].

Regarding the MPPD model, the estimations made for MWNT-7 deposition and retention from the aerosol characterization reported by Umeda et al. [66] closely approximated the real measurements relayed by Kasai et al. [45] as well as our own data [39] (Fig. 5). Other estimations for Graphistrength C100, Nanocyl NC7000, or NM-403 appear quite efficient and relevant. It is nevertheless obvious that improvements are still needed to improve prediction. How the aspect ratio for CNT and fibre-like aerosols - which is of paramount importance in deposition and retention - is taken into account deserves particular attention. Likewise, the clearance module, and more specifically parameter adjustment, will also need to be improved. Studies such as the one presented here integrating an adequate (if not exhaustive) characterization of the aerosols as well as measurements taken at various post-exposure times should provide useful data for those seeking to improve existing models.

## Conclusion

The results presented in this article demonstrate the correlation, in both the short-term and the long-term, between inflammation (evaluated by measuring % PMN in BALF) and the surface area dose retained within the lung following acute to subchronic inhalation of three class of NMs: TiO_2_, CBs (both representing PSLT), and MWCNTs (representing HARN). The relationship between inflammation and retained surface area dose takes the form of a sigmoid curve whatever the exposure duration. The equation fitting the curve depends on the class of NM considered. Based on the surface area dose retained, most MWCNTs clearly exhibited a higher inflammatory potential than PSLT. Thus, a retained surface dose of 5 cm^2^/g lung was sufficient to trigger an inflammatory response with MWCNTs, whereas it was necessary to reach overload (or quasi overload) conditions with PSLT before neutrophil infiltration was measured. These conditions corresponded to retained surface area doses greater than 150 cm^2^/g lung.

The surface area dose is a useful metric for hazard grouping. This metric also made it possible to distinguish two categories of MWCNTs, or rather to specify the geometric limits of what is usually designated as long and thick, or short and thin tubes which would present very distinct toxicological profiles (the former being much more toxic than the latter). According to our observations, any nanotube measuring several hundred nm long with an aspect ratio exceeding 20–25 should be considered long and thick and potentially harmful. In addition, by using surface area as dose metric it becomes possible to account for surface-induced toxicity for both micrometric and nanometric materials.

The fact that – for a given material type and exposure scenario - %PMN closely correlates with retained surface area dose regardless of post-exposure time has important implications for human health as it suggests that - at least for those types of materials - bioprocessing has neither a mitigating nor an aggravating effect on the surface-specific inflammogenicity of these materials in the lung. Thus, long-term pulmonary inflammation due to inhaled particles (e.g. urban dust) can be reliably predicted for humans using publicly available dosimetry models (e.g. MPPD) combined occupational and/or ambient exposure data.

This work only considered data relating to three classes of materials; it would now deserve to be extended to include other material types and morphologies (including some porous materials).

Once sufficient data has been acquired, the inflammatory potential of a substance and its longer-term consequences could be assessed by estimating the surface area dose retained based on the BET surface area of a powder, and its aerosol parameters (rigorously determined following a well-defined aerosol characterization strategy). Using this type of approach would significantly reduce the use of animals.

## Supplementary Information

**Additional file 1 **: **Supplemental 1.** Inhalation set-up for P25 and Printex-90 aerosol exposures. **Supplemental 2.** Inhalation set-up for MWNT-7 aerosols exposure. **Supplemental 3.** Representative transmission electron microscopy images of (A) P25, (B) Printex-90 and (C) MWNT-7 aerosols. **Supplemental 4.** Number (left panel) and mass (right panel) particle size distributions of (A) agglomerated P25, (B) nebulized P25, (C) Printex-90, and (D) MWNT-7 aerosols. **Supplemental 5.** Cytology of bronchoalveolar lavage fluid (left lung) and lung and body weights for control animals and rats exposed to the different aerosols (*n* = 6 per group). **Supplemental 6.** Lung burden and clearance of P25 TiO_2_ (normalized to the airborne TiO_2_ concentration and fitted with a first order kinetic model) for the different exposure conditions (RBG: dry powder; AGK: dried nebulized suspension of powder). **Supplemental 7.** Lung burden and clearance of Printex-90 CB (normalized to airborne CB concentration) for the different exposure conditions (dry powder). **Supplemental 8.** Details of the post-exposure times for TiO_2_ material-induced effects on neutrophil influx as a function of retained surface area dose for different exposure times as depicted in Fig. 2: 1-week (triangle), 2-week (diamond), 4-week (square) or 13-week (circle). Both nanoparticles (unicolor) and fine particles (bicolor) were considered. **Supplemental 9.** Details of the post-exposure times for CB-induced effects on neutrophil influx as a function of retained surface area dose for different exposure times as depicted in Fig. 4: 4-week (or 6-week for Monarch 880 and Elftex 12) (square) or 13-week (circle). **Supplemental 10.** Details of the post-exposure times for MWCNT-induced effects on neutrophil influx as a function of retained surface area dose for different exposure times as depicted in Fig. 5: 1-week (triangle), 2-week (diamond), 4-week (square), or13-week (circle). Open symbols indicate that the retained surface area dose was estimated.

## Data Availability

The datasets used and/or analyzed during the current study are available from the corresponding author on reasonable request.

## Abbreviations

AO: Adverse outcome
AOP: Adverse outcome pathway
APS: Aerodynamic particle sizer
BAL: Bronchoalveolar lavage
BALF: Bronchoalveolar fluid
BET: Brunauer-Emmett-Teller
CB: Carbon black
CFC: Closed-face cassette
CMD: Count median diameter
CMoAD: Count modal aerodynamic diameter
COPD: Chronic obstructive pulmonary disease
CPC: Condensation particle counter
D: Day
ELPI: Electrical low-pressure impactor
FRC: Functional residual capacity
HARN: High aspect ratio nanomaterial
HO: Head-only
GSD: Geometric standard deviation
KE: Key event
MMAD: Mass median aerodynamic diameter
MPPD: Multiple-Path Particle Dosimetry
MWCNT: Multiwall carbon nanotubes
NM: Nanomaterial
NO: Nose-only
OECD: Organisation for Economic Co-operation and Development
OPC: Optical particle counter
PMN: Polymorphonuclear neutrophil
PSLT: Poorly soluble particles of low toxicity
RBG: Rotating brush generator
SD: Standard deviation
SMPS: Scanning mobility particle sizer
TG: Test guideline
TiO_2_: Titanium dioxide
URT: Upper respiratory tract volume
W: Week
WB: Whole-body
