# Supplementary material for: Retained particle surface area dose drives inflammation in rat lungs following acute, subacute, and subchronic inhalation of nanomaterials
Source: Part Fibre Toxicol. 2021 Aug 5;18:29. doi: 10.1186/s12989-021-00419-w (PMC8340536; doi:10.1186/s12989-021-00419-w)

**Supplemental 1**

Inhalation set-up for P25 and Printex-90 aerosol exposures


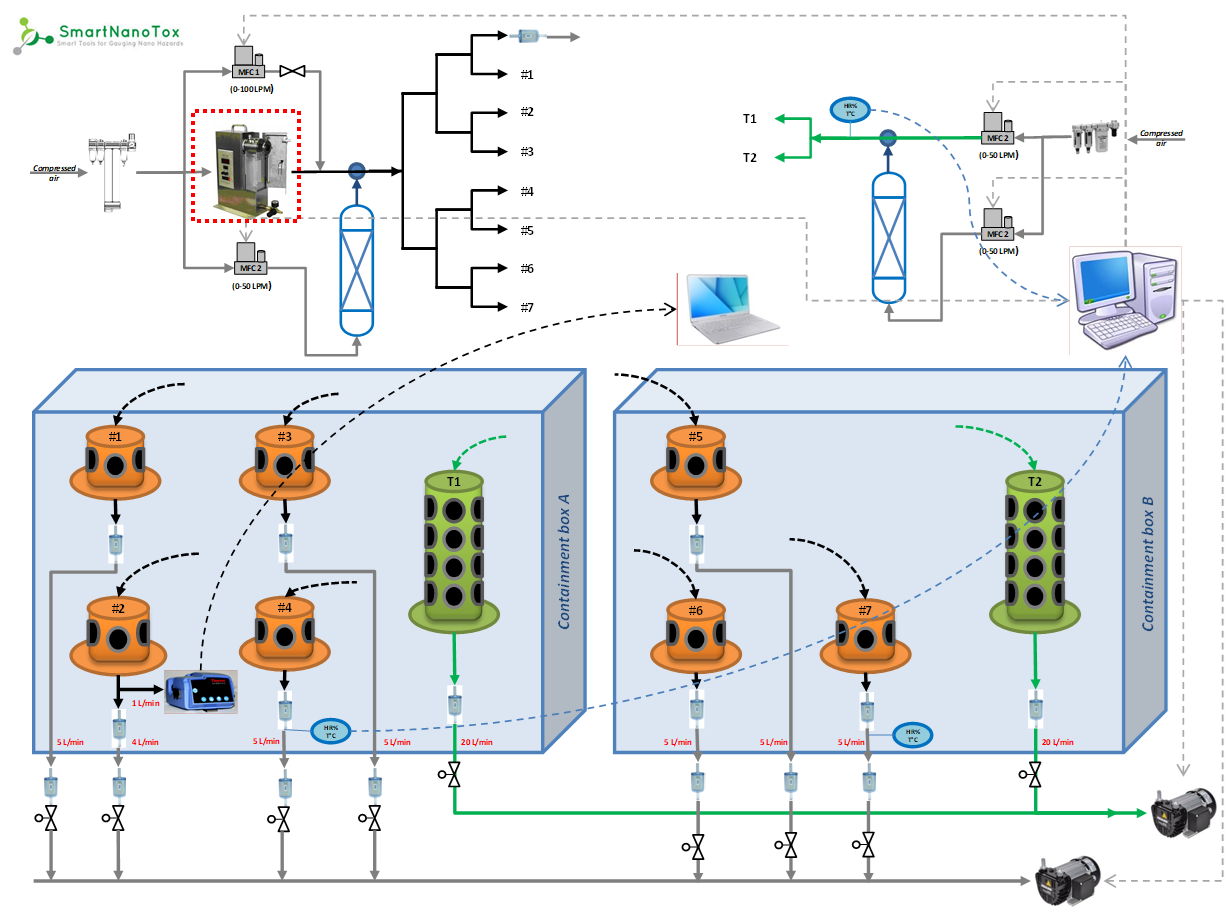


*Rotating brush aerosol generator (RBG1000 in red dotted rectangle) used to generate dry TiO_2_ P25 aerosols. It was replaced by a SAG410/U solid aerosol generator for CB Printex-90 aerosol generation, or by 2 nebulizers (AGK2000) for P25 aerosol generation from water suspensions.*

**Supplemental 2**

Inhalation set-up for MWNT-7 aerosols exposure


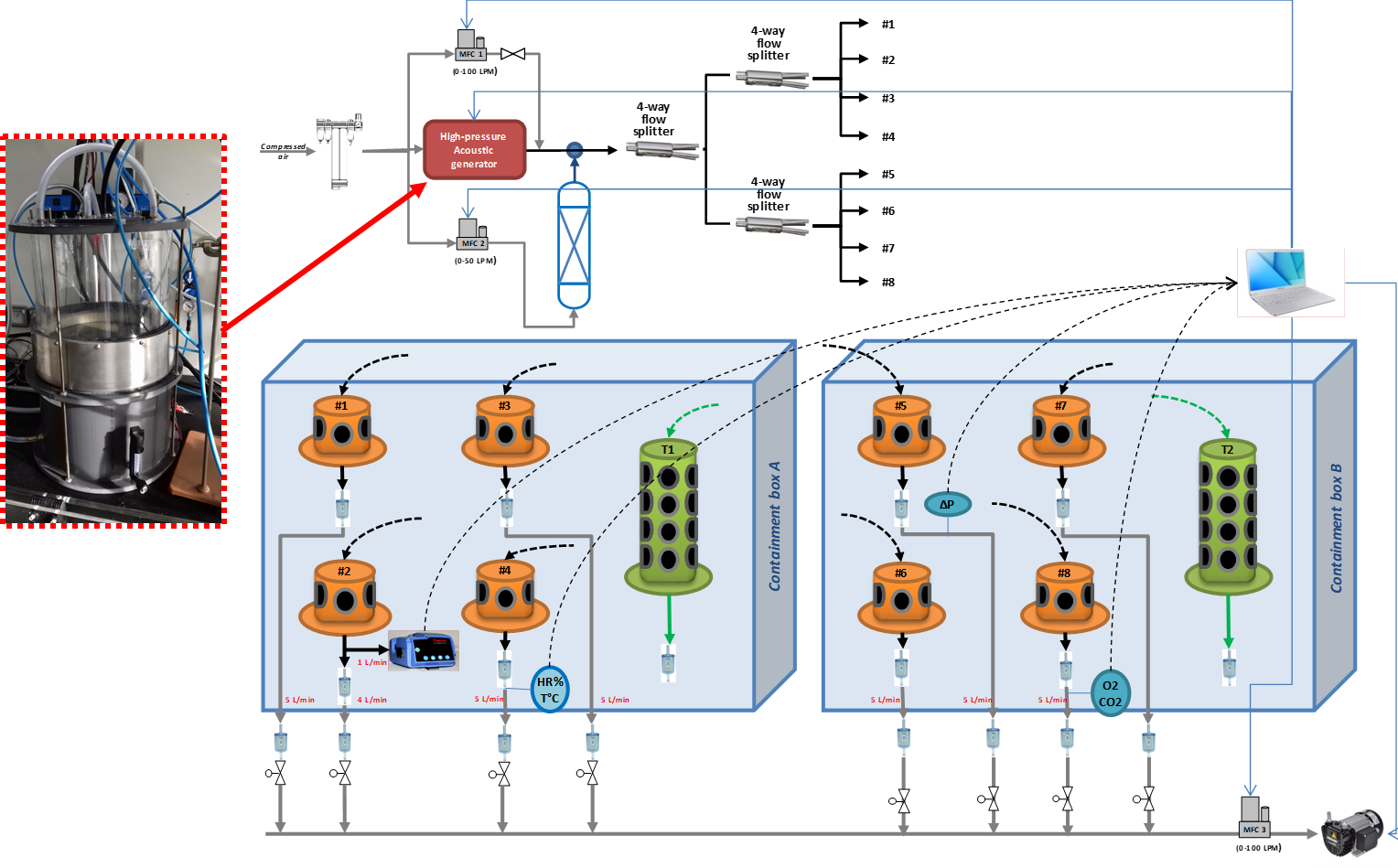


**Supplemental 3**

Representative transmission electron microscopy images of (A) P25, (B) Printex-90 and (C) MWNT-7 aerosols.

Aerosols were directly collected on TEM grids (using a mini particle sampler, MPS Ecomesure) and observed under a Philips Transmission electron microscope equipped with a Gatan digital camera.

| A   | B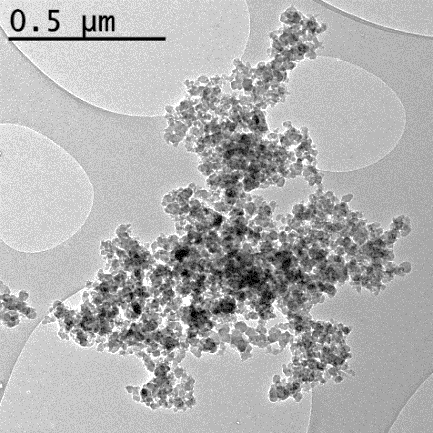 | C   |
| --- | --- | --- |

**Supplemental 4**

Number (left panel) and mass (right panel) particle size distributions of (A) agglomerated P25, (B) nebulized P25, (C) Printex-90, and (D) MWNT-7 aerosols.

| A  **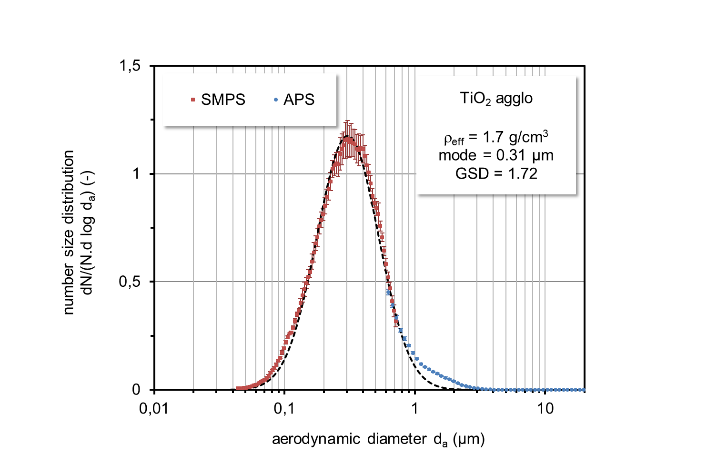** | 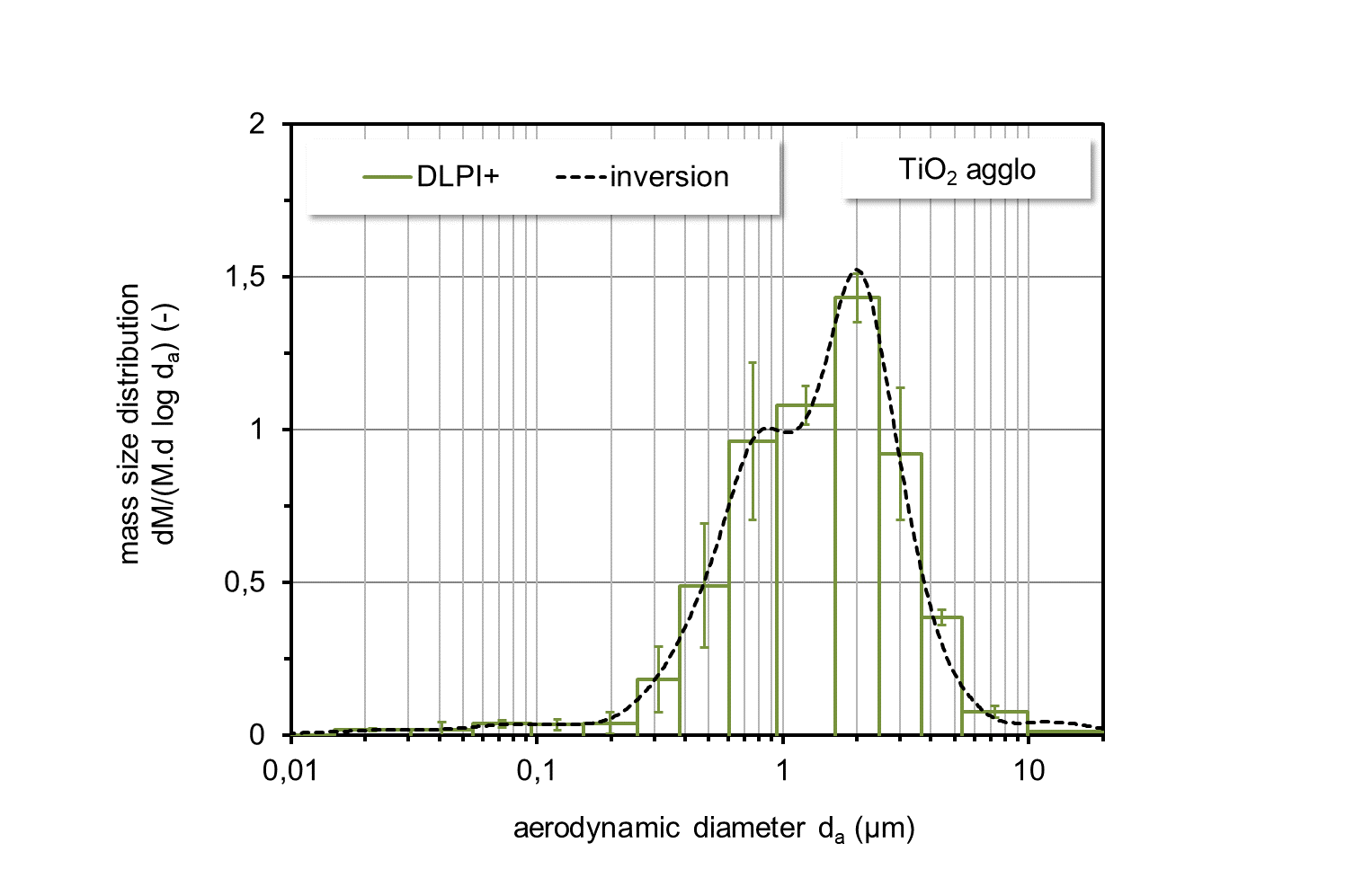 |
| --- | --- |
| B  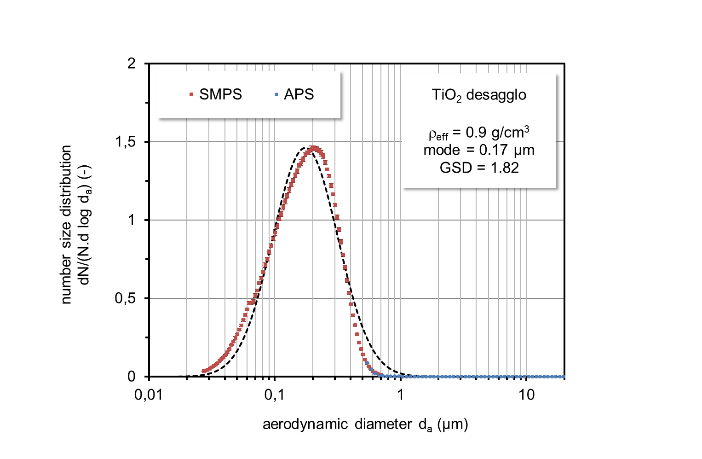 | 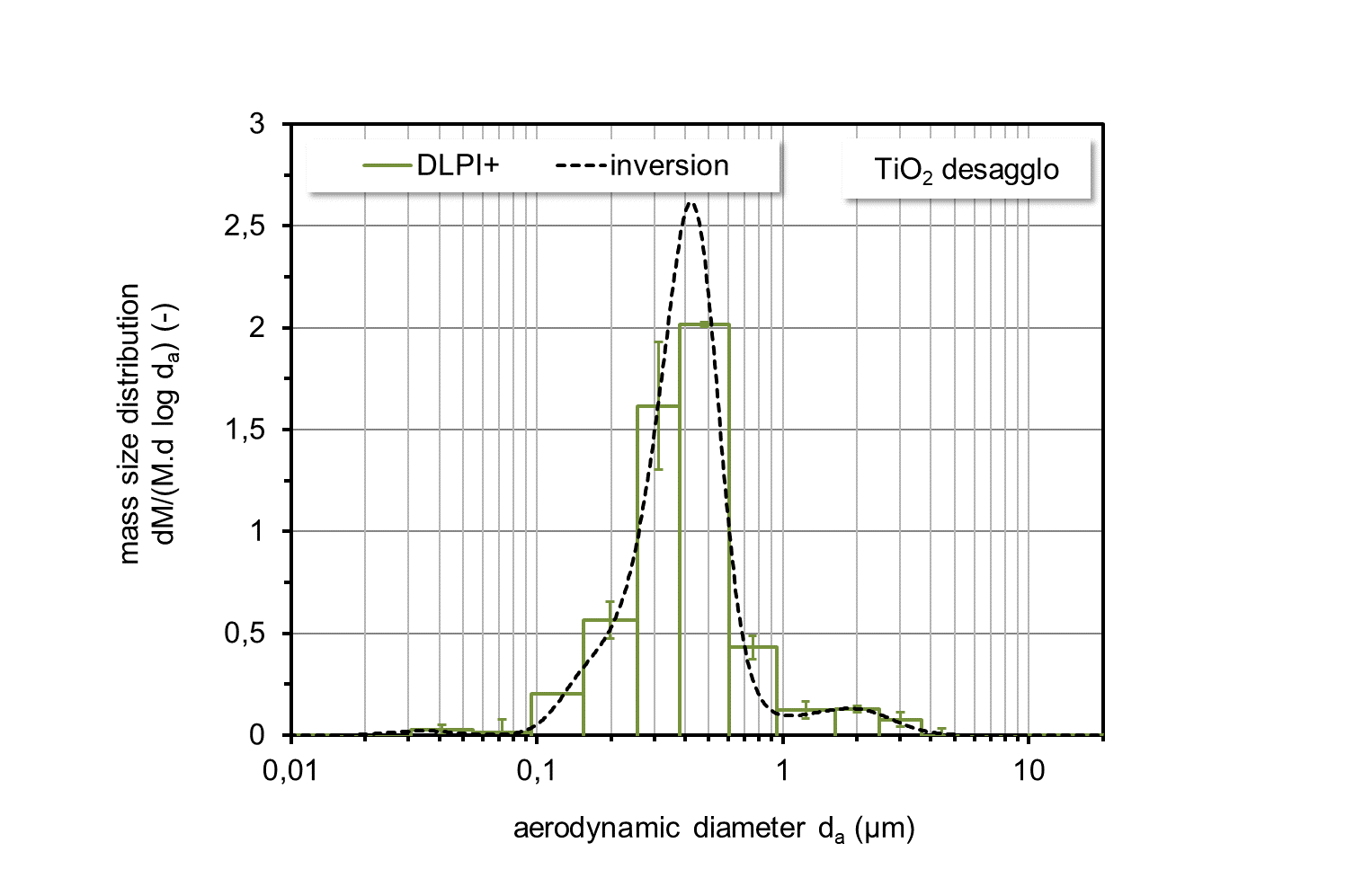 |
| C  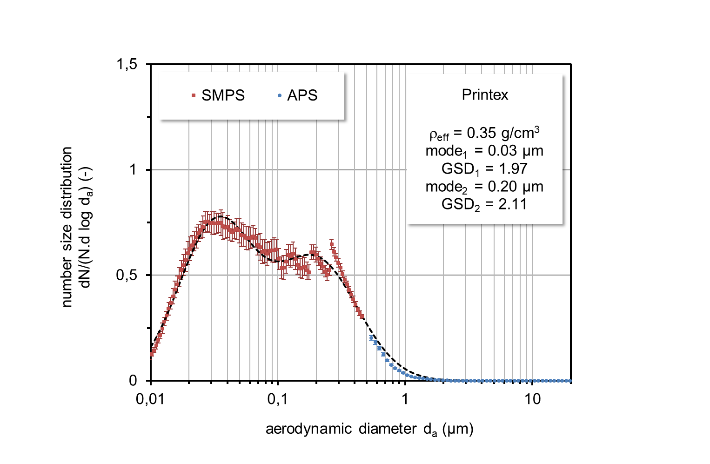 | 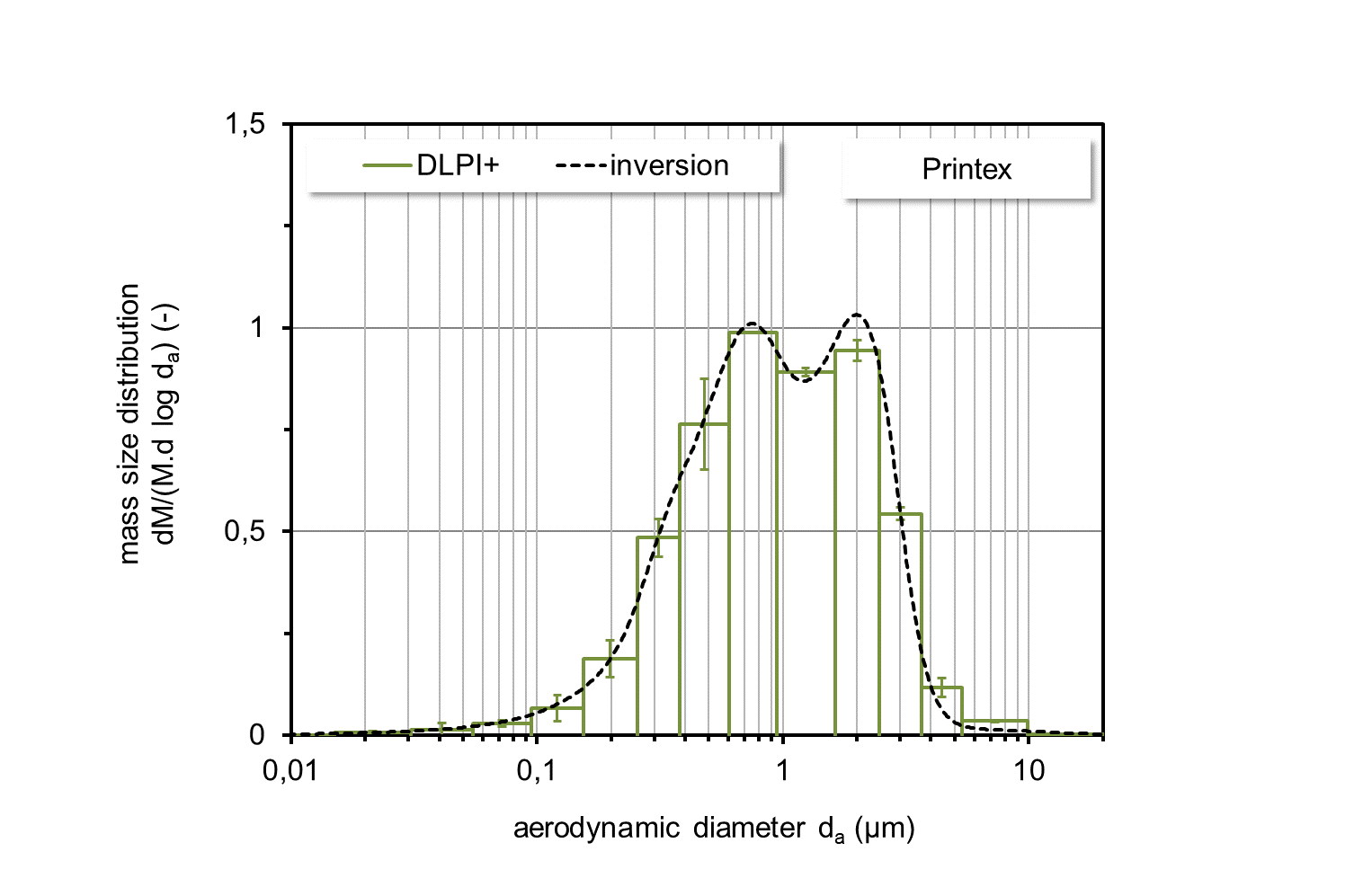 |
| D  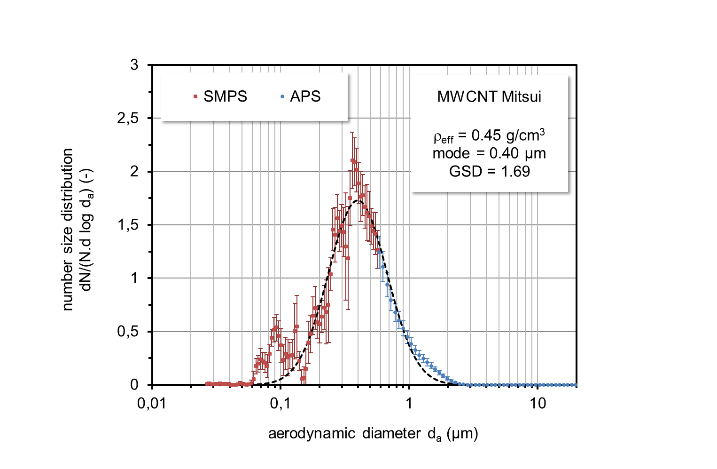 | 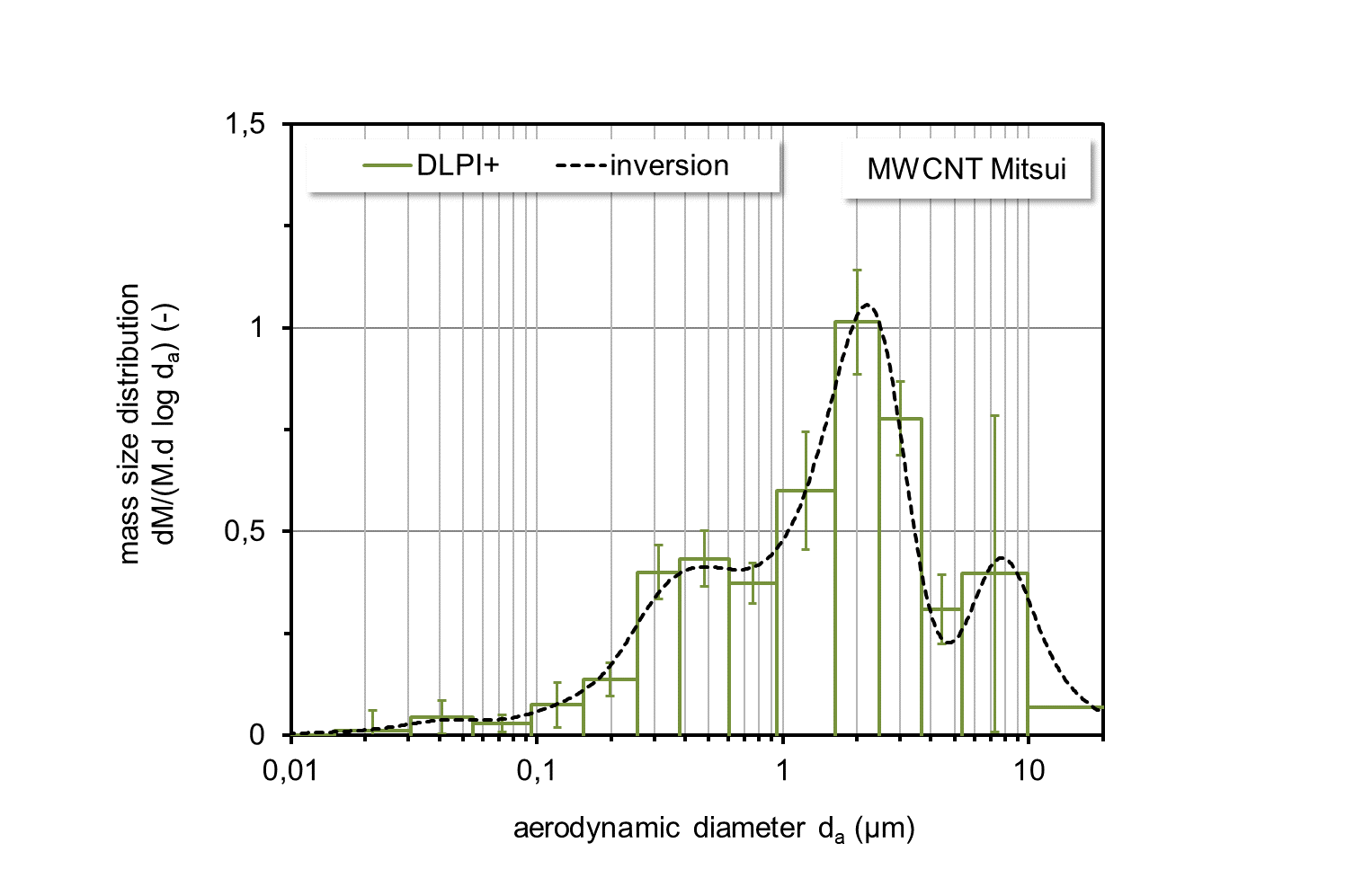 |

**Supplemental 5**

Cytology of bronchoalveolar lavage fluid (left lung) and lung and body weights for control animals and rats exposed to the different aerosols (n = 6 per group).

| **Material** | **Day** | **Target concentration**  **(mg/m^3^)** | **Neutrophils**  **×10^4^/lobe** | **Macrophages**  **×10^4^/lobe** | **Lymphocytes**  **×10^3^/lobe** | **Total cells**  **×10^4^/lobe** | **Lung weight**  **(g)** | **Body weight**  **(g)** |
| --- | --- | --- | --- | --- | --- | --- | --- | --- |
| P25 | D3 | 0 | 0.3±0.2 | 40.6±17.5 | 0.5±0.8 | 40.9±17.4 | 1.33±0.08 | 291.5±22.7 |
|  |  | 1.5^a^ | 1.1±1.0 | 45.3±10.8 | 2.3±2.4 | 46.7±10.4 | 1.38±0.10 | 292.5±13.9 |
|  |  | 5^a^ | 6.4±2.9 | 40.2±10.5 | 1.9±2.5 | 46.8±12.3 | 1.44±0.07 | 291.7±14.7 |
|  |  | 15 | 53.2±6.9 | 78.5±20.6 | 3.8±2.5 | 132.1±20.9 | 1.59±0.11 | 291.3±14.8 |
|  | W4 | 0 | 0.7±0.6 | 44.8±11.6 | 0.6±0.7 | 45.6±11.6 | 1.36±0.07 | 338.5±17.7 |
|  |  | 1.5^a^ | 0.8±0.1 | 32.4±7.2 | 1.2±1.4 | 33.1±6.5 | 1.44±0.14 | 332.5±31.3 |
|  |  | 5^a^ | 4.6±4.0 | 29.2±6.9 | 1.3±0.7 | 28.7±2.0 | 1.39±0.10 | 329.9±19.0 |
|  |  | 15 | 52.0±20.8 | 43.5±29.1 | 5.3±5.0 | 90.7±46.7 | 1.56±0.16 | 325.9±20.7 |
|  | W26 | 0 | 0.7±0.7 | 75.1±34.2 | 1.0±2.0 | 77.0±36.4 | 1.48±0.13 | 385.9±37.8 |
|  |  | 1.5^a^ | 1.3±1.4 | 76.0±33.9 | 0.4±0.9 | 77.3±33.3 | 1.55±0.14 | 401.3±22.0 |
|  |  | 5^a^ | 1.4±1.3 | 74.4±16.2 | 0.6±1.5 | 75.9±16.9 | 1.48±0.10 | 376.7±39.7 |
|  |  | 15 | 20.9±14.9 | 78.6±10.1 | 1.5±1.7 | 99.7±15.6 | 1.62±0.10 | 386.5±28.3 |
| P25^b^ | D0 | 0 | 1.6±1.9 | 75.2±10.7 | 0.9±1.3 | 76.8±11.3 | 1.21±0.08 | 260.6±10.3 |
|  |  | 5 | 3.9±0.9 | 85.7±15.2 | 1.0±1.1 | 89.7±15.7 | 1.18±0.06 | 254.3±18.1 |
|  | D3 | 0 | 1.1±0.5 | 85.5±7.5 | 0.2±0.6 | 86.6±7.2 | 1.22±0.05 | 290.1±16.0 |
|  |  | 5 | 7.8±2.2 | 89.0±10.0 | 1.3±1.6 | 96.9±9.6 | 1.18±0.06 | 261.9±12.8 |
|  | W4 | 0 | 1.5±0.9 | 111.3±29.1 | 1.9±0.9 | 112.9±29.6 | 1.32±0.05 | 338.0±17.2 |
|  |  | 5 | 1.4±0.6 | 116.2±8.8 | 1.4±0.9 | 117.7±8.9 | 1.35±0.06 | 350.4±20.9 |
|  | W13 | 0 | 0.8±0.5 | 113.2±21.2 | 1.6±1.8 | 114.2±21.7 | 1.34±0.13 | 359.3±20.2 |
|  |  | 5 | 1.0±0.4 | 113.4±46.3 | 0.2±0.4 | 114.4±46.4 | 1.44±0.12 | 380.0±41.8 |
|  | W26 | 0 | 1.1±0.3 | 90.0±20.3 | 2.9±2.2 | 91.3±20.4 | 1.59±0.07 | 445.1±19.7 |
|  |  | 5 | 1.5±0.7 | 78.3±13.7 | 3.8±3.1 | 80.2±14.1 | 1.51±0.13 | 427.6±33.2 |
| Printex90 | D3 | 0 | 0.3±0.4 | 40.3±5.9 | 0.5±0.8 | 41.9±6.3 | 1.29±0.10 | 209.6±8.8 |
|  |  | 5^a^ | 11.2±4.0 | 49.8±20.8 | 1.0±1.8 | 61.2±23.4 | 1.37±0.06 | 305.4±20.9 |
|  |  | 15^a^ | 71.0±30.3 | 51.3±10.4 | 4.9±7.0 | 122.8±26.5 | 1.61±0.19 | 295.1±13.7 |
|  |  | 50 | 124.1±13.5 | 46.2±11.3 | 1.3±3.1 | 170.5±16.9 | 1.91±0.07 | 298.0±12.1 |
|  | W4 | 0 | 1.2±1.7 | 40.4±15.6 | 0.0±0.0 | 41.6±15.2 | 1.38±0.13 | 322.6±20.9 |
|  |  | 5^a^ | 1.9±0.5 | 35.0±6.2 | 0.3±0.4 | 36.9±5.9 | 1.50±0.05 | 325.4±11.6 |
|  |  | 15^a^ | 25.4±2.5 | 43.1±6.0 | 0.0±0.0 | 74.9±16.4 | 1.54±0.08 | 338.4±11.8 |
|  |  | 50 | 62.8±39.9 | 54.4±28.6 | 0.3±0.8 | 117.2±66.7 | 1.77±0.17 | 331.2±22.9 |
|  | W26 | 0 | 0.7±0.6 | 69.7±19.0 | 2.4±2.2 | 70.6±19.2 | 1.47±0.06 | 366.0±34.2 |
|  |  | 5^a^ | 1.3±1.1 | 69.0±31.6 | 1.7±2.7 | 70.5±31.6 | 1.56±0.13 | 372.6±22.7 |
|  |  | 15^a^ | 8.5±7.2 | 69.4±14.2 | 3.2±2.4 | 78.2±21.4 | 1.51±0.10 | 359.2±23.7 |
|  |  | 50 | 70.8±29.3 | 79.4±44.5 | 13.6±24.8 | 151.5±74.0 | 1.92±0.21 | 378.4±28.9 |
| MWNT-7 | D3 | 0 | 1.8±2.2 | 40.9±15.0 | 0.0±0.0 | 42.8±16.4 | 1.30±0.05 | 270.8±12.7 |
|  |  | 0.15^a^ | 2.3±1.1 | 31.0±10.2 | 0.4±1.1 | 34.9±12.0 | 1.42±0.10 | 296.3±22.7 |
|  |  | 0.5^a^ | 14.0±10.2 | 29.7±7.7 | 0.5±1.2 | 43.8±14.1 | 1.60±0.14 | 290.2±12.3 |
|  |  | 1.5 | 30.3±13.2 | 35.9±6.9 | 0.0±0.0 | 66.2±14.8 | 1.67±0.09 | 292.9±7.4 |
|  | D30 | 0 | 0.9±0.7 | 43.8±7.3 | 0.8±1.2 | 44.7±6.9 | 1.39±0.14 | 318.8±13.9 |
|  |  | 0.15^a^ | 0.3±0.2 | 33.0±4.0 | 0.4±0.6 | 35.1±5.9 | 1.44±0.09 | 327.3±16.3 |
|  |  | 0.5^a^ | 3.2±1.2 | 33.9±4.8 | 0.9±1.0 | 37.2±4.2 | 1.51±0.10 | 314.8±11.9 |
|  |  | 1.5 | 16.0±6.1 | 40.7±12.3 | 1.6±3.5 | 56.8±14.0 | 1.69±0.11 | 322.1±13.5 |
|  | D180 | 0 | 0.4±0.4 | 56.7±13.7 | 0.1±0.2 | 64.2±25.1 | 1.56±0.06 | 359.9±21.9 |
|  |  | 0.15^a^ | 2.8±2.8 | 55.1±19.4 | 0.2±0.4 | 57.9±19.7 | 1.54±0.09 | 365.0±28.0 |
|  |  | 0.5^a^ | 4.8±5.6 | 45.8±13.5 | 0.3±0.4 | 50.6±18.0 | 1.60±0.08 | 353.1±20.9 |
|  |  | 1.5 | 6.4±2.1 | 76.3±51.4 | 1.1±1.3 | 82.8±52.9 | 1.68±0.10 | 352.2±29.6 |

*^a^ 6h-equivalent concentrations created by modulating the exposure time to the aerosols*

*^b^ aerosol produced from a suspension by nebulization*

**Supplemental 6**

Lung burden and clearance of P25 TiO_2_ (normalized to the airborne TiO_2_ concentration and fitted with a first order kinetic model) for the different exposure conditions (RBG: dry powder; AGK: dried nebulized suspension of powder).

Normalization (to airborne concentration) allowed the incidence of airborne concentration on the deposition efficiency to be addressed. In case of lung overload, the normalized lung burden tended to decrease with increasing airborne concentration.


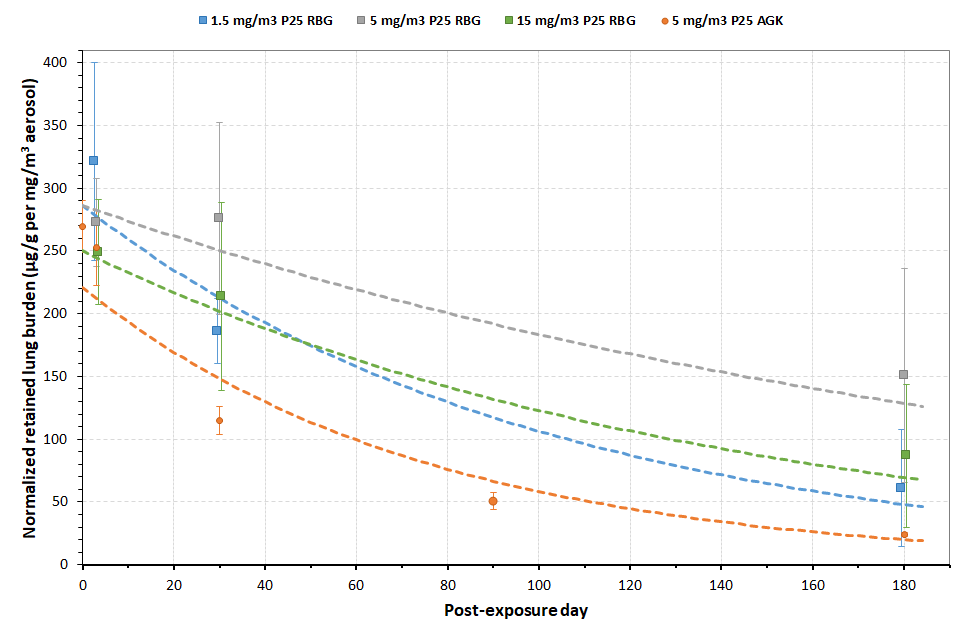
Equations:

- 1.5 mg/m^3^ P25 RBG : y = exp(5.6568-0.0099x); R² = 0.776; t_1/2_ = 70 days
- 5 mg/m^3^ P25 RBG : y = exp(5.657-0.0044x) ; R² = 0.408; t_1/2_ = 156 days
- 15 mg/m^3^ P25 RBG : y = exp(5.521-0.0071x) ; R² = 0.588; t_1/2_ = 98 days
- 5 mg/m^3^ P25 AGK : y = exp(4.8040-0.0134x) ; R² = 0.936; t_1/2_ = 52 days

**Supplemental 7**

Lung burden and clearance of Printex-90 CB (normalized to airborne CB concentration) for the different exposure conditions (dry powder).

Normalization (to airborne concentration) allowed the incidence of airborne concentration on the deposition efficiency to be addressed. The decrease in normalized retained lung burden with increasing Printex-90 airborne concentration reflects lung overload.


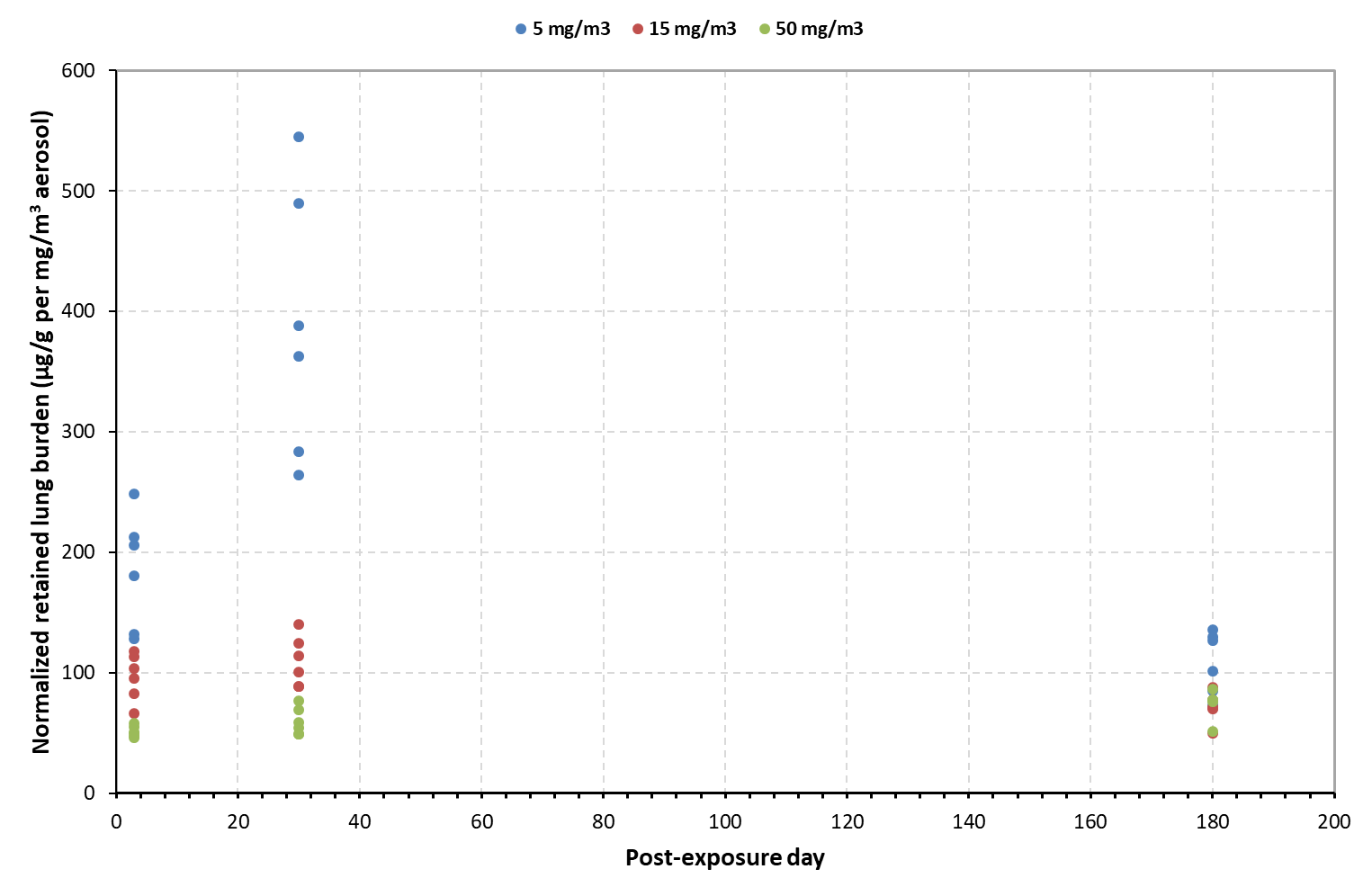


**Supplemental 8**

Details of the post-exposure times for TiO_2_ material-induced effects on neutrophil influx as a function of retained surface area dose for different exposure times as depicted in Figure 2: 1-week (triangle), 2-week (diamond), 4-week (square) or 13-week (circle). Both nanoparticles (unicolor) and fine particles (bicolor) were considered.


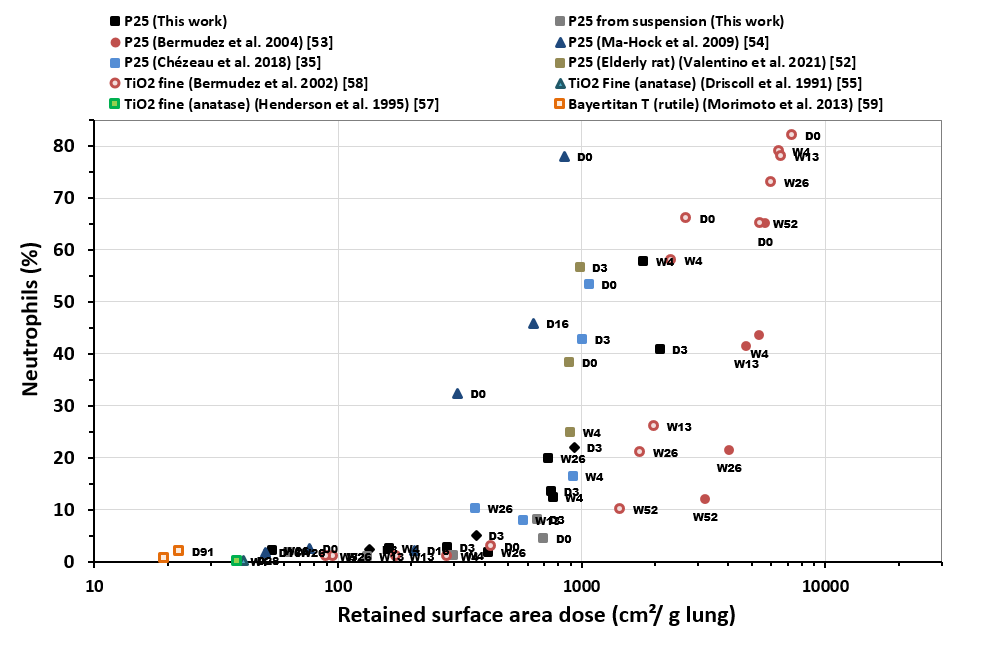


**Supplemental 9**

Details of the post-exposure times for CB-induced effects on neutrophil influx as a function of retained surface area dose for different exposure times as depicted in Figure 4: 4-week (or 6-week for Monarch 880 and Elftex 12) (square) or 13-week (circle).


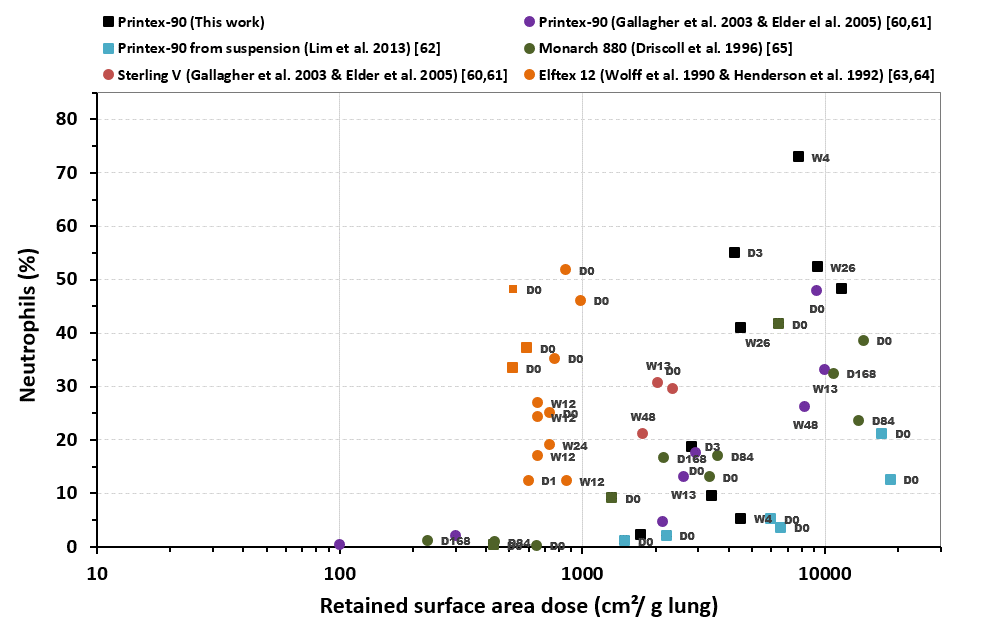


**Supplemental 10**

Details of the post-exposure times for MWCNT-induced effects on neutrophil influx as a function of retained surface area dose for different exposure times as depicted in Figure 5: 1-week (triangle), 2-week (diamond), 4-week (square), or13-week (circle). Open symbols indicate that the retained surface area dose was estimated.


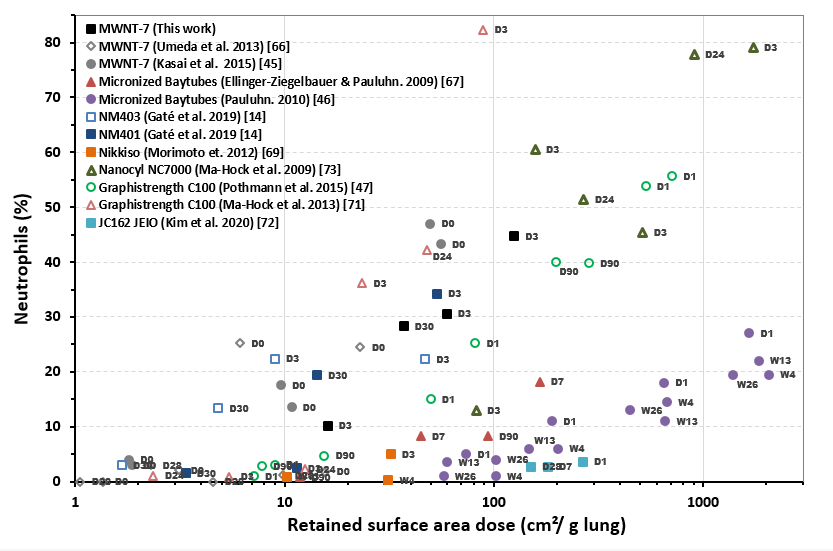

Supplement: Supplementary file 1 — Additional file 1 : Supplemental 1. Inhalation set-up for P25 and Printex-90 aerosol exposures. Supplemental 2. Inhalation set-up for MWNT-7 aerosols exposure. Supplemental 3. Representative transmission electron microscopy images of (A) P25, (B) Printex-90 and (C) MWNT-7 aerosols. Supplemental 4. Number (left panel) and mass (right panel) particle size distributions of (A) agglomerated P25, (B) nebulized P25, (C) Printex-90, and (D) MWNT-7 aerosols. Supplemental 5. Cytology of bronchoalveolar lavage fluid (left lung) and lung and body weights for control animals and rats exposed to the different aerosols (n = 6 per group). Supplemental 6. Lung burden and clearance of P25 TiO2 (normalized to the airborne TiO2 concentration and fitted with a first order kinetic model) for the different exposure conditions (RBG: dry powder; AGK: dried nebulized suspension of powder). Supplemental 7. Lung burden and clearance of Printex-90 CB (normalized to airborne CB concentration) for the different exposure conditions (dry powder). Supplemental 8. Details of the post-exposure times for TiO2 material-induced effects on neutrophil influx as a function of retained surface area dose for different exposure times as depicted in Fig. 2: 1-week (triangle), 2-week (diamond), 4-week (square) or 13-week (circle). Both nanoparticles (unicolor) and fine particles (bicolor) were considered. Supplemental 9. Details of the post-exposure times for CB-induced effects on neutrophil influx as a function of retained surface area dose for different exposure times as depicted in Fig. 4: 4-week (or 6-week for Monarch 880 and Elftex 12) (square) or 13-week (circle). Supplemental 10. Details of the post-exposure times for MWCNT-induced effects on neutrophil influx as a function of retained surface area dose for different exposure times as depicted in Fig. 5: 1-week (triangle), 2-week (diamond), 4-week (square), or13-week (circle). Open symbols indicate that the retained surface area dose was estimated. [file 12989_2021_419_MOESM1_ESM.docx]
